# PTBP1 Regulates DNMT3B Alternative Splicing by Interacting With RALY to Enhance the Radioresistance of Prostate Cancer

**DOI:** 10.1002/advs.202405997

**Published:** 2024-09-17

**Authors:** Haixia He, Qianghua Zhou, Yangjie Zhang, Yi Li, Lin Ding, Ting Shen, Sen Liu, Shengmeng Peng, Ming Huang, Hua Zhou, Liang Cheng, Ruihui Xie, Qiang Zhang, Junlin Lu, Liting Li, Jing Yang, Shoumin Bai, Tianxin Lin, Xu Chen

**Affiliations:** ^1^ Department of Radiation Oncology Sun Yat‐sen Memorial Hospital Sun Yat‐sen University Guangzhou 510120 China; ^2^ Department of Urology Sun Yat‐sen Memorial Hospital Sun Yat‐sen University Guangzhou 510120 China; ^3^ Guangdong Provincial Key Laboratory of Malignant Tumor Epigenetics and Gene Regulation Sun Yat‐Sen Memorial Hospital Sun Yat‐Sen University Guangzhou 510120 China; ^4^ Department of Urology Sun Yat‐sen University Cancer Center Guangzhou 510060 China; ^5^ State Key Laboratory of Oncology in South China & Collaborative Innovation Center of Cancer Medicine Sun Yat‐sen University Cancer Center Guangzhou 510060 China; ^6^ Department of Urology Pu'er People's Hospital of Yunnan Province Pu'er 665000 China

**Keywords:** DNMT3B, prostate cancer, PTBP1, radioresistance, splicing factor

## Abstract

Radiotherapy is a curative arsenal for prostate cancer (PCa), but radioresistance seriously compromises its effectiveness. Dysregulated RNA splicing factors are extensively involved in tumor progression. Nonetheless, the role of splicing factors in radioresistance remains largely unexplored in PCa. Here, 23 splicing factors that are differentially expressed between PCa and adjacent normal tissues across multiple public PCa databases are identified. Among those genes, polypyrimidine tract binding protein 1 (PTBP1) is significantly upregulated in PCa and is positively associated with advanced clinicopathological features and poor prognosis. Gain‐ and loss‐of‐function experiments demonstrate that PTBP1 markedly reinforces genomic DNA stability to desensitize PCa cells to irradiation in vitro and in vivo. Mechanistically, PTBP1 interacts with the heterogeneous nuclear ribonucleoproteins (hnRNP) associated with lethal yellow protein homolog (RALY) and regulates exon 5 splicing of DNA methyltransferase 3b (DNMT3B) from DNMT3B‐S to DNMT3B‐L. Furthermore, upregulation of DNMT3B‐L induces promoter methylation of dual‐specificity phosphatase‐2 (DUSP2) and subsequently inhibits DUSP2 expression, thereby increasing radioresistance in PCa. The findings highlight the role of splicing factors in inducing aberrant splicing events in response to radiotherapy and the potential role of PTBP1 and DNMT3B‐L in reversing radioresistance in PCa.

## Graphical abstract

PTBP1 is overexpressed in prostate cancer and is associated with poor prognosis. PTBP1 markedly reinforced genomic DNA stability to desensitize prostate cancer cells to irradiation. Mechanistically, PTBP1 binds to RALY and together regulates alternative splicing of DNMT3B to increase DNMT3B‐L accumulation, leading to hypermethylation of the DUSP2 promoter and downregulation of its expression, and then increasing radioresistance of prostate cancer.

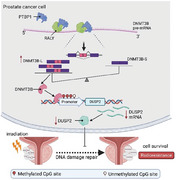

## 1 Introduction

Ionizing radiation efficiently eliminates tumor cells by causing genomic DNA damage. Thus, radiotherapy has become a widely used genotoxic cell‐killing treatment for various cancers, particularly prostate cancer (PCa), which is the second leading cause of tumor‐related mortality in men.^[^ ^1^ ^]^ Notably, radiotherapy is a curative treatment for localized PCa and has also been approved as adjuvant therapy after surgery, salvage therapy following biochemical recurrence, and even palliative care in the presence of distant metastasis.^[^ ^2^ ^]^ Unfortunately, despite its success in the treatment of PCa, nearly 30–50% of patients still suffer from long‐term relapse or short‐term disease progression within primary or metastatic sites due to the emergence of radiation resistance. Therefore, there is an urgent need to elucidate the molecular mechanisms underlying radiation resistance.^[^ ^3^, ^4^ ^]^

Alternative splicing (AS) is a tightly regulated post‐transcriptional cellular process that increases protein diversity in eukaryotes. Statistically, over 95% of human genes undergo AS, and cancers exhibit up to 30% more AS events than normal tissues do, which contributes to diverse gene variants involved in tumorigenesis.^[^ ^5^, ^6^, ^7^ ^]^ Typically, AR splice variant 7 (AR‐V7), a truncated isoform of the AR‐FL protein, persistently activates the AR signaling pathway and subsequently induces resistance to androgen deprivation therapy in PCa.^[^ ^8^, ^9^ ^]^ Generally, the regulation of AS primarily depends on *trans*‐acting splicing factors that specifically recognize and bind intrinsic *cis* elements.^[^ ^10^ ^]^ Aberrant expression of these splicing factors in multiple human malignancies leads to splicing reprogramming which facilitates tumor progression and therapeutic resistance including radioresistance.^[^ ^11^ ^]^ For example, serine and arginine rich splicing factor 1 (SRSF1), a splicing factor, has been shown to induce radioresistance in lung cancer cells by modulating the AS of protein tyrosine phosphatase mitochondrial 1 (PTPMT1).^[^ ^12^ ^]^ Therefore, specifically targeting dysregulated splicing factors may open novel avenues for cancer treatment. Nevertheless, it remains unclear whether aberrant AS and the corresponding splicing factors are involved in the radioresistance of PCa.

In this study, we screened for potential splicing factors that contribute to PCa progression on the basis of multiple public databases and identified increased expression of polypyrimidine tract‐binding protein 1 (PTBP1) in PCa tissues. PTBP1 commonly binds to polypyrimidine sequences to form loops in the corresponding regions of pre‐mRNAs to prevent the assembly of the splicing complex, leading to the inhibition of AS.^[^ ^13^ ^]^ However, PTBP1 can also activate splicing events by antagonizing other splicing repressors, such as SRp30c.^[^ ^14^ ^]^ In addition to its role in AS regulation, PTBP1 participates in RNA polyadenylation, maturation, and translation as well.^[^ ^15^, ^16^ ^]^ Here, through in vivo and in vitro experiments, we confirmed that PTBP1 promotes exon 5 inclusion of DNA methyltransferase 3b (DNMT3B) with the assistance of the heterogeneous nuclear ribonucleoproteins (hnRNP) associated with lethal yellow protein homolog (RALY), which subsequently drives radioresistance in PCa. Our findings highlight the therapeutic potential of targeting PTBP1 to sensitize PCa patients to radiotherapy.

## 2 Results

### 2.1 Identification of PTBP1 as an Upregulated Splicing Regulator Closely Associated with PCa Progression

**Figure 1:**
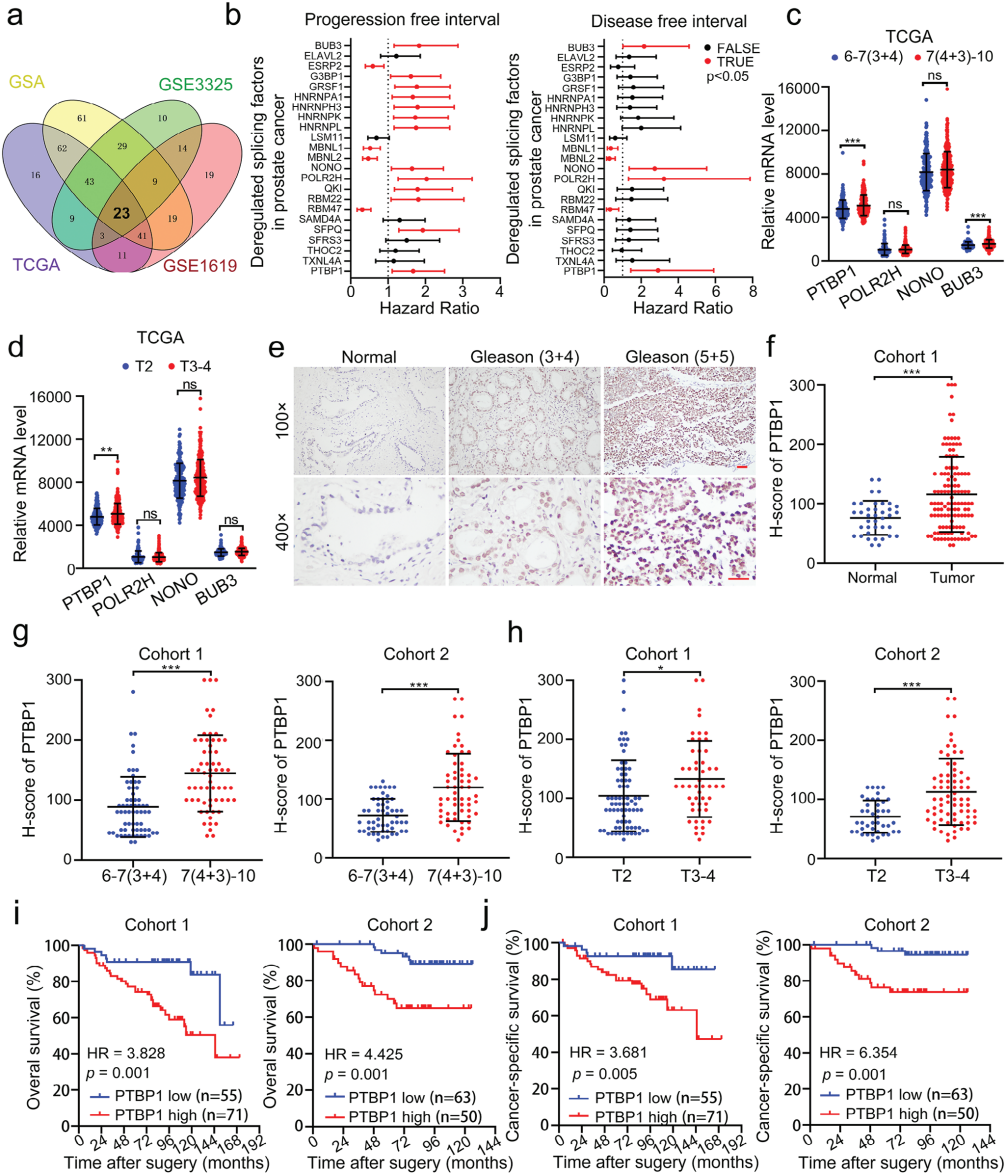
Elevated PTBP1 expression is closely associated with poor prognosis in prostate cancer. a) Venn diagram showing the overlapping splicing factors with dysregulated expression in prostate cancer (PCa) across the indicated public databases. GSA: Genome Sequence Archive, GEO: Gene Expression Omnibus database. TCGA: The Cancer Genome Atlas. b) Forrest plots of hazard ratios for the associations between 23 dysregulated splicing factors and progression‐free intervals (left) and disease‐free intervals (right) in the TCGA database. Prognostic splicing factors (red) with *p* <0.05 were considered statistically significant. c) Difference in the expression of PTBP1, POLR2H, NONO and BUB3 between low Gleason score (6‐7(3+4)) PCa tissues and high Gleason score (7(4+3)−10) PCa tissues in the TCGA database. d) Difference in the expression of PTBP1, POLR2H, NONO and BUB3 between low T stage (T2) PCa tissues and high T stage (T3‐4) PCa tissues in the TCGA database. e) Representative immunohistochemical images of PTBP1 in high and low Gleason score PCa tissues and normal tissues. Scale bar, 50 µm. f) Difference in the expression of PTBP1 between PCa tissues and normal tissues in cohort 1. g) Difference in the expression of PTBP1 between low Gleason score (6‐7(3+4)) PCa tissues and high Gleason score (7(4+3)−10) PCa tissues from cohort 1(left) and cohort 2 (right). h) Difference in the expression of PTBP1 between low T stage (T2) PCa tissues and high T stage (T3‐4) PCa tissues from cohort 1(left) and cohort 2 (right). i) Kaplan–Meier curves for the overall survival of PCa patients with high or low expression of PTBP1 in Cohort 1 (left) and Cohort 2 (right). j) Kaplan–Meier curves for the cancer‐specific survival of PCa patients with high or low expression of PTBP1 in Cohort 1 (left) and Cohort 2 (right). *p < 0.05, **p < 0.01, ***p < 0.001, ns. no significance. Student's *t* test.

To identify dysregulated splicing factors related to PCa progression, we first analyzed the expression levels of 374 known splicing factors in PCa tissues compared with those in normal tissues from The Cancer Genome Atlas (TCGA), Gene Expression Omnibus (GEO), and Genome Sequence Archive (GSA) databases.^[^ ^17^ ^]^ A total of 23 splicing factors were dysregulated in PCa tissues, of which 15 genes were upregulated and 8 genes were downregulated (**Figure** **1**a). We subsequently estimated the prognostic significance of these 23 dysregulated splicing factors via univariate Cox proportional hazards regression analysis based on data from the TCGA database. As shown in Figure 1b, BUB3, NONO, POLR2H, and PTBP1 were significantly associated with a worse progression‐free interval (PFI) and disease‐free interval (DFI), while MBNL1, MBNL2, and RBM47 were significantly correlated with an extended PFI and DFI. Moreover, correlation analysis demonstrated that, out of the four splicing regulators associated with unfavorable prognosis, only PTBP1 exhibited significant correlations with advanced T stage and Gleason score (Figure 1c,d). Thus, PTBP1 was selected for further investigation. We further assessed PTBP1 expression in PCa tissues and adjacent normal tissues from 239 cases in two cohorts (cohort 1 and cohort 2) using Immunohistochemistry (IHC) staining (Table S1, Supporting Information). Consistently, PTBP1 was overexpressed in PCa tissues compared with adjacent normal tissues (Figure 1e,f). Further analysis demonstrated that high expression of PTBP1 was also significantly associated with advanced Gleason score and T stage (Figure 1g,h). Kaplan–Meier survival analysis revealed that patients with higher levels of PTBP1 had shorter overall survival (OS) and cancer‐specific survival (CSS) in both cohorts (Figure 1i,j). Univariate Cox proportional hazards regression analysis revealed that high PTBP1 expression conferred a greater than two‐fold increase in the risk of mortality, especially cancer‐specific death, in both cohorts (OS: cohort 1, HR = 3.25, 95% CI = 1.48–7.15, *p* = 0.003; cohort 2, HR = 4.26, 95% CI = 1.73–11.35, *p* = 0.002; CSS: cohort 1, HR = 2.39, 95% CI = 1.08–5.30, *p* = 0.031; and cohort 2, HR = 6.35, 95% CI = 1.79–22.60, *p* = 0.004; Table S2, Supporting Information). Furthermore, multivariate analysis indicated that high PTBP1 expression was an independent prognostic factor for OS in cohort 1 and for CSS in both cohorts (OS: cohort 1, HR = 2.72, 95% CI = 1.04–7.09, *p* = 0.041; CSS: cohort 1, HR = 3.47, 95% CI = 1.06–11.34, *p* = 0.040; cohort 2, HR = 4.37, 95% CI = 1.14–16.79, *p* = 0.032; Table S3, Supporting Information). Taken together, these results demonstrate that PTBP1 overexpression is associated with unfavorable clinicopathological features and serves as a worse prognostic factor in PCa patients.

### 2.2 PTBP1 Enhances the Proliferation and Radiotherapy Tolerance of PCa Cells In Vitro

To ascertain the functional role of PTBP1 in PCa cells, we established DU145 and PC‐3 cells with PTBP1 knockdown (KD) or overexpression (OE) via PTBP1‐targeted siRNAs or lentiviral transfection (Figure S1a,b, Supporting Information). The results of the CCK‐8 assay demonstrated that PTBP1 KD resulted in a pronounced inhibitory effect on the cell viability of PCa cells, whereas PTBP1 OE exhibited the opposite effects (Figure S1c,d, Supporting Information). Moreover, the proliferation‐promoting function of PTBP1 was reinforced by the colony formation assay (Figure S1e,f, Supporting Information). In parallel, the results of the EdU incorporation assays illustrated that PTBP1 played a significant role in regulating the cell population in the S phase (Figure S1g,h, Supporting Information). These suggest that PTBP1 may promote proper progression to the DNA duplication phase of the cell cycle.

**Figure 2:**
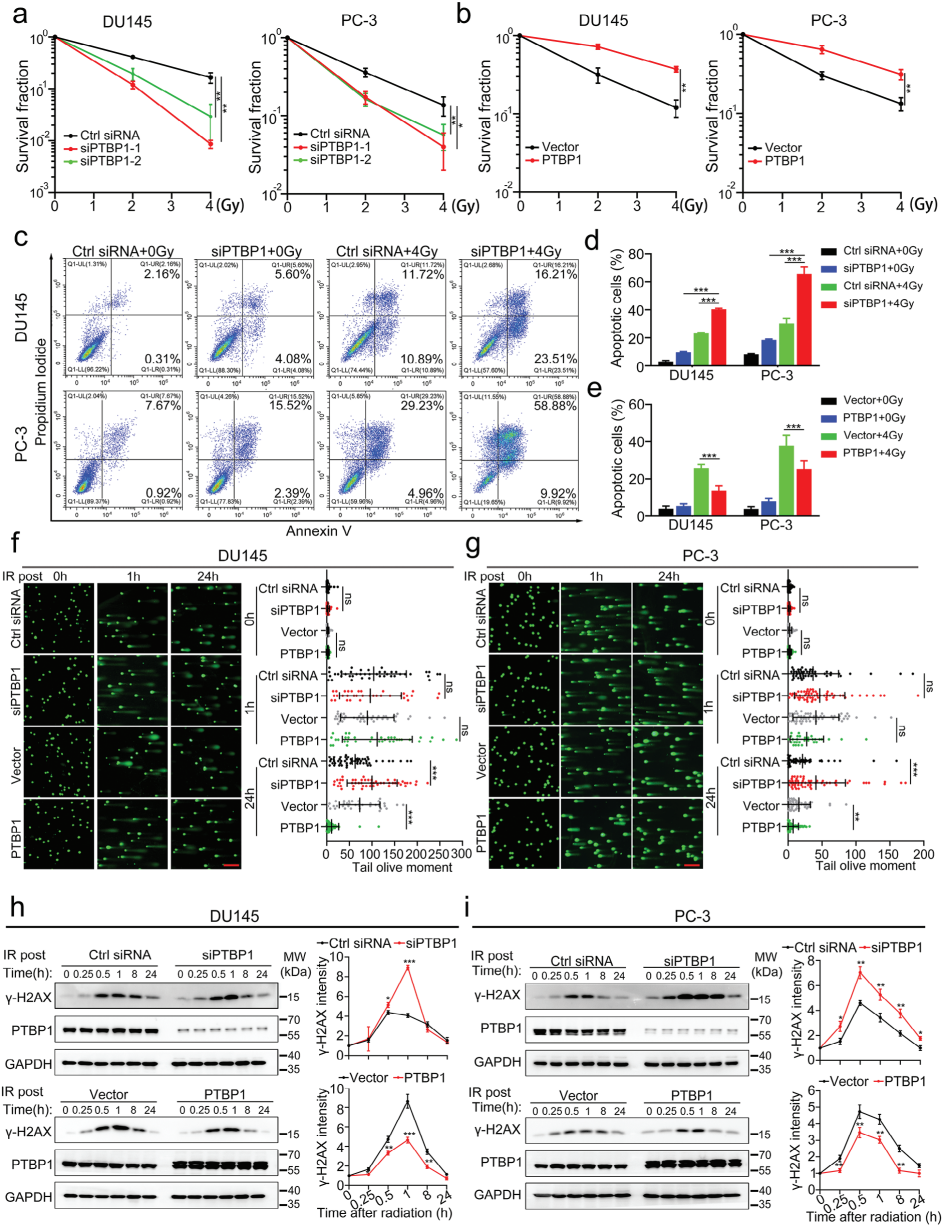
PTBP1 facilitates prostate cancer cell resistance to radiotherapy by maintaining genomic stability a) Clonogenic survival in response to irradiation (0, 2, and 4 Gy) of prostate cancer (PCa) cells transiently transfected with the indicated siRNAs. b) Clonal survival of PCa cells transfected with PTBP1 or control plasmids in response to irradiation (0, 2, and 4 Gy). c,d) Images (c) and quantification (d) of cell apoptosis of PCa cells transfected with the indicated siRNAs and exposed to 4 Gy irradiation. e) Quantification of the cell apoptosis of PCa cells transfected with PTBP1 or control plasmids and exposed to 4 Gy irradiation. f,g) Representative images (left) and statistical analyses (right) of the comet assay results of the indicated DU145 (f) and PC‐3 (g) cells at 0, 1, and 24 h after 4 Gy irradiation. Scale bar, 20 µm. h,i) Protein levels of γ‐H2AX in the indicated DU145 (h) and PC‐3 (i) cells at the indicated times after 4 Gy irradiation were measured via western blot (left) and gray quantitative analysis (right). The data are presented as the means ± S.D.s of three independent experiments. *p < 0.05, **p < 0.01, ***p < 0.001 by two‐tailed Student's *t* test or by two‐way ANOVA followed by Tukey's post‐hoc test where applicable. ns. no significance.

It is reported that PTBP1 is involved in DNA damage repair, which is one of the principal causes of radiotherapy resistance.^[^ ^18^ ^]^ To investigate the potential involvement of PTBP1 in response to irradiation (IR), PCa cells with PTBP1 KD or OE were treated with various doses of IR. Clonogenic assays demonstrated that PTBP1 KD significantly reduced the colony formation of PCa cells after IR, whereas PTBP1 OE increased the resistance of PCa cells to IR treatment (**Figure** **2**a,b; Figure S2a, Supporting Information). Moreover, when exposed to 4 Gy of IR, PTBP1 KD markedly increased the proportion of apoptotic PCa cells compared with that in the control group (Figure 2c,d). Reciprocally, the survival potential of PCa cells with PTBP1 OE was greater than that of control cells after 4 Gy IR (Figure 2e; Figure S2b, Supporting Information). To further investigate whether PTBP1 affects IR‐induced genomic instability, a comet assay was performed to examine DNA damage in PTBP1 KD or OE PCa cells at 0, 1, and 24 h post‐IR. Compared with control PCa cells, PTBP1‐KD PCa cells presented much longer comet tails, but PTBP1‐OE PCa cells presented shorter comet tails at 24 h after IR treatment (Figure 2f,g). In parallel, WB assay revealed that the levels of the γ‐H2AX protein were increased in PTBP1 KD PCa cells, and decreased in PTBP1 OE PCa cells, compared with their respective control PCa cells at various time points after 4 Gy IR treatment (Figure 2h,i). These results indicate that PTBP1 plays a significant role in promoting PCa cell proliferation and resistance to radiation therapy.

### 2.3 PTBP1 Overexpression Reinforced Radiation Resistance of PCa Cells In Vivo

**Figure 3:**
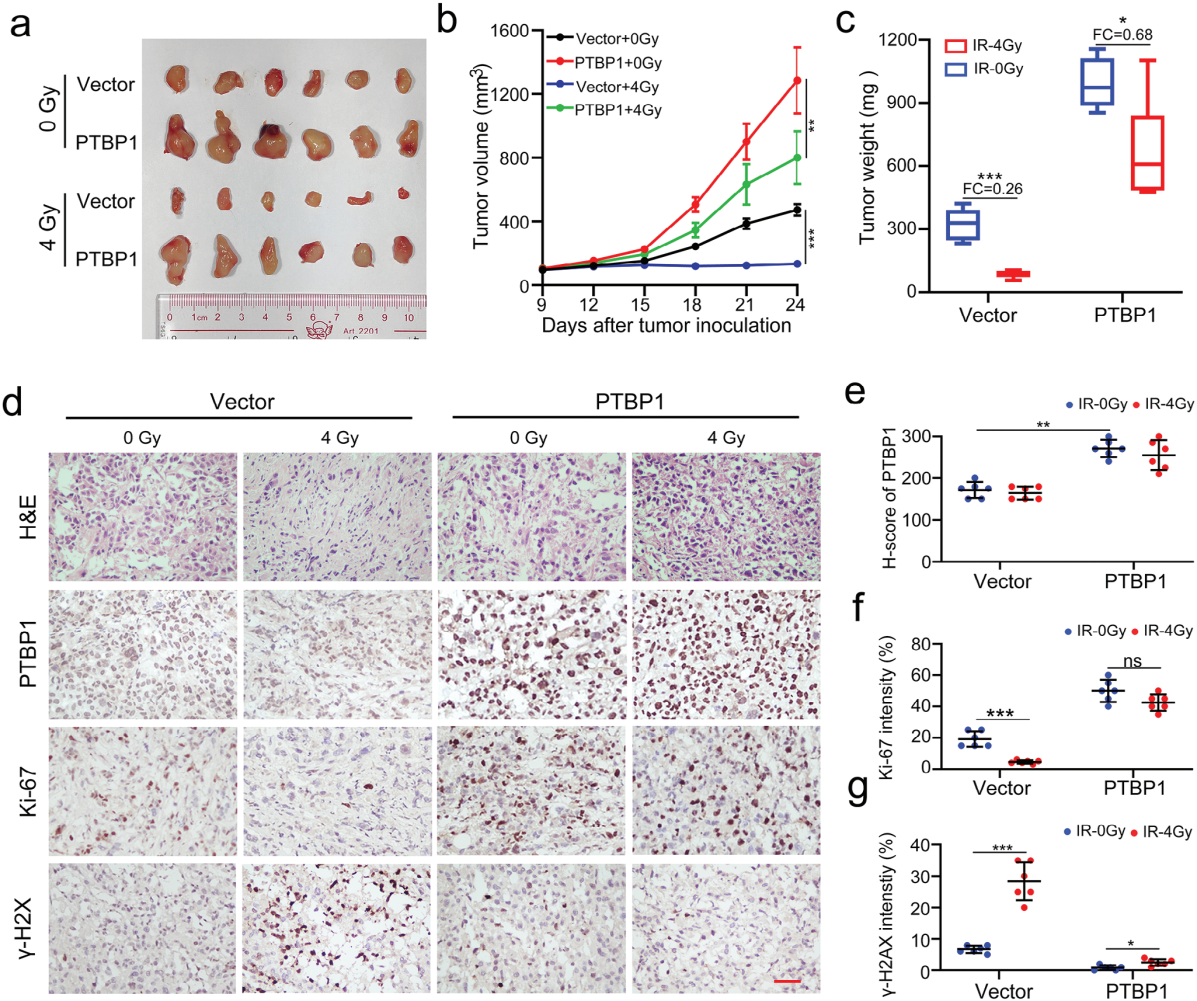
Overexpression of PTBP1 increases radioresistance of prostate cancer cells in vivo. a) Representative image of xenograft tumors in the indicated groups treated with or without 4 Gy irradiation. b) Tumor volume curves in the indicated groups following irradiation treatment, measured every 3 days. c) Statistical analysis of the tumor volume in the indicated groups. d) Representative images of hematoxylin‐eosin and immunohistochemical staining of PTBP1, ki‐67 and γ‐H2AX in xenograft tumors from the indicated groups; Scale bar, 50 µm. e–g) Statistical analyses of difference in PTBP1 (e), Ki‐67 (f) and γ‐H2AX (g) expression among the indicated groups. The data are presented as the mean ± S.D.s of three independent experiments. *p < 0.05, **p < 0.01, ***p < 0.001 by two‐tailed Student's *t* test, or by two‐way ANOVA followed by Tukey's post‐hoc test where applicable. ns. no significance.

To validate whether the in vitro findings were applicable in vivo, a xenograft mouse model was established by subcutaneously implanting treated PC‐3 cells into nude mice. After 9 days, subcutaneous tumors developed and were randomly irradiated (4 Gy) twice at 1‐week intervals. The growth of vector PC‐3 tumors was significantly attenuated after IR treatment, whereas PTBP1 OE resulted in a greater rate of tumor growth than control, causing a less pronounced reduction in xenograft volume and weight post‐IR (**Figure** **3**a–c). Consistent with the cell‐based results, the IHC staining revealed that a reduction in Ki‐67 expression only appeared in the vector PC‐3 tumors after IR treatment (Figure 3d–f). Moreover, after IR treatment, γ‐H2AX expression was largely increased in the vector group, but only weakly upregulated in the PTBP1 OE group (Figure 3g). Collectively, these findings indicate that PTBP1 is essential for the radioresistance of PCa.

### 2.4 PTBP1 Induces DNMT3B Exon 5 Inclusion Through Direct Binding to Pre‐mRNAs

**Figure 4:**
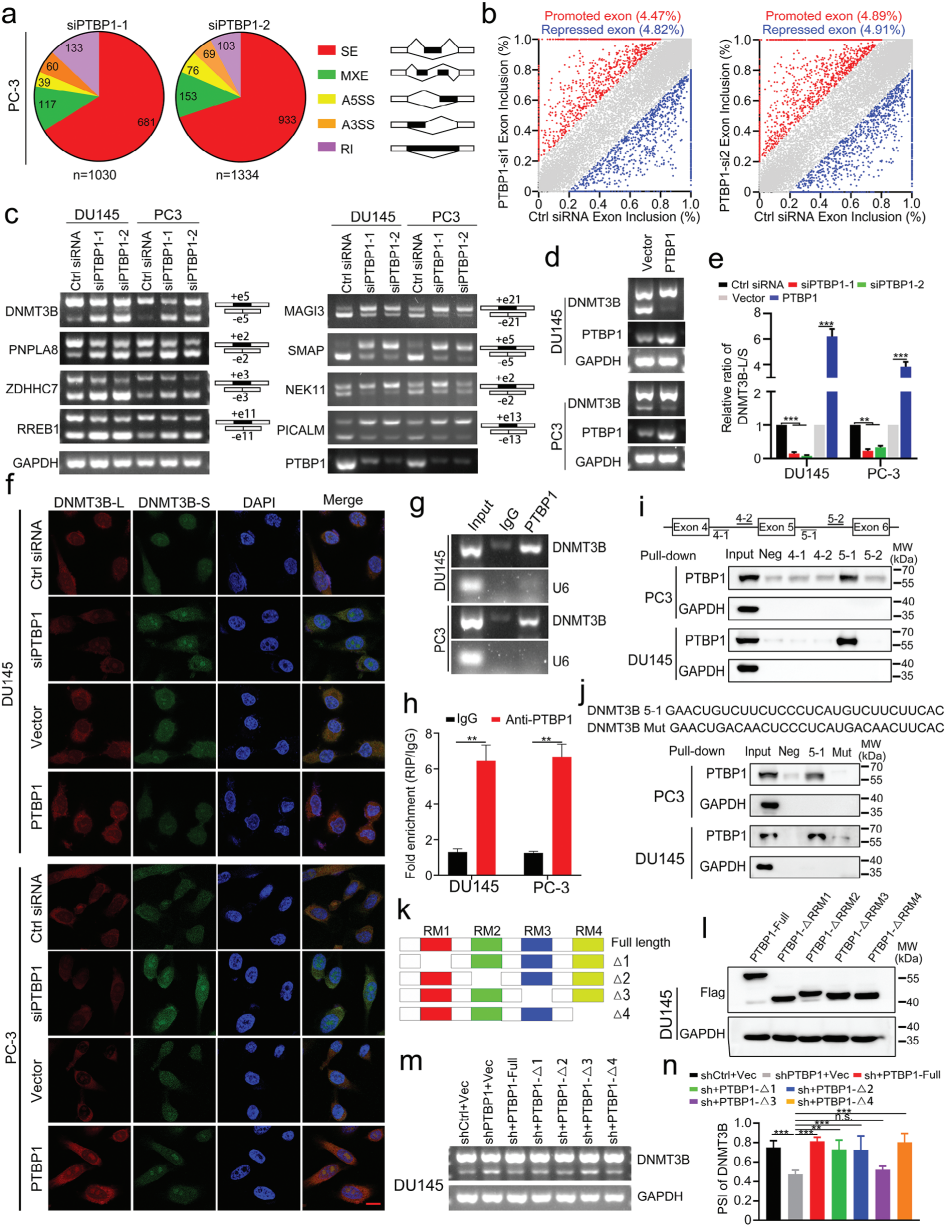
PTBP1 mediates the inclusion of exon 5 in DNMT3B pre‐mRNA. a) Quantitative analysis of the different alternative splicing events, including five categories, after PC‐3 cells were transfected with the indicated siRNAs. b) Scatterplots showing PTBP1 knockdown with the indicated siRNAs affecting skipped exon events. c) Intron exclusion (left) or inclusion (right) of candidate genes in PTBP1 knockdown prostate cancer (PCa) cells was validated by agarose gel electrophoresis of PCR products. d) Representative gel electrophoresis image of DNMT3B intron retention in PTBP1 overexpressing PCa cells. e) RT‐PCR‐based statistical analysis of the DNMT3B‐L/DNMT3B‐S ratio in PTBP1 knockdown or PTBP1 overexpressing PCa cells. f) Representative images of RNA‐FISH images showing the changes in DNMT3B‐L and DNMT3B‐S in PTBP1‐knockdown or PTBP1‐overexpressing PCa cells. Scale bar, 20 µm. g,h) Representative images (g) and statistical analysis (h) of DNMT3B pre‐mRNA enrichment on PTBP1 from the RIP assay in PCa cells using anti‐PTBP1 antibody. i) RNA‐pulldown assay followed by western blotting demonstrating the interaction of PTBP1 with the 4 indicated intron segments of DNMT3B pre‐mRNA. j Specific interactions of PTBP1 with 5‐1 intron segments of DNMT3B pre‐mRNA were validated by RNA pulldown assay followed by western blotting. K,l) Schematic diagram (k) and protein expression (l) of PTBP1 truncation variants lacking RRM1, RRM2, RRM3, or RRM4. m,n) Representative image (m) and statistical analysis (n) of RT‐PCR results of DNMT3B‐L/DNMT3B‐S PSI in DU145 cells with PTBP1 knockdown, and re‐overexpression of PTBP1 deletion mutants. The data are presented as the means ± S.D.s of three independent experiments. *p < 0.05, **p < 0.01, ***p < 0.001 by two‐tailed Student's *t* test, or by two‐way ANOVA followed by Tukey's post‐hoc test where applicable. ns. no significance.

To dissect the molecular mechanism by which PTBP1 promotes radioresistance in PCa, we first investigated whether PTBP1 modulates the AS of MESI2, PKM, and AXL, all of which have been reported to regulate tumor progression.^[^ ^19^ ^]^ Unexpectedly, the AS of these genes was not regulated by PTBP1, suggesting that other alternative genes are involved in PTBP1‐mediated radioresistance in PCa (Figure S3a, Supporting Information). Thus, we conducted RNA‐seq analysis to investigate AS events regulated by PTBP1 in PC‐3 cells with or without PTBP1 KD. A total of 1030 and 1334 PTBP1‐regulated AS events were identified in two independent siRNAs, which were categorized into 5 AS categories (**Figure** **4**a). Among these events, skipped exon (SE) events were the most affected, indicating that PTBP1 mainly modulates SEs (Figure 4a). Subsequent analysis indicated the dual role of PTBP1 as both a splicing activator and repressor, as it induced similar percentages of exon inclusion and exclusion events (Figure 4b). Among all PTBP1‐mediated SEs, 78 genes were initially selected on the basis of FDR<0.05 and |IncLevelDifference| > 0.2 for both siRNAs. Next, 22 genes were excluded due to false positives, and 56 genes were ultimately identified. To further verify the accuracy of our RNA‐seq findings, the top 8 PTBP1‐affected AS events were validated via PCR, as shown in Figure 4c. These results further confirmed that PTBP1 either activated or repressed the splicing of the targeted genes. Among the validated PTBP1‐affected AS events, we selected DNMT3B, since it is one of the genes whose expression is the most significantly altered upon PTBP1 KD and has a broad effect on tumor progression, especially radioresistance.^[^ ^20^, ^21^, ^22^, ^23^ ^]^ Consistently, PTBP1 OE led to an obvious increase in DNMT3B‐L inclusion of exon 5 in PCa cells (Figure 4d,e). RNA‐FISH also demonstrated that PTBP1 KD obviously diminished the transcript level of DNMT3B‐L, but increased the mRNA level of DNMT3B‐S, whereas PTBP1 OE had the opposite effect (Figure 4f).

To determine whether PTBP1‐induced exon retention was mediated by direct binding to the pre‐mRNA of DNMT3B, a RNA Immunoprecipitation (RIP) assay was performed. Intriguingly, we found that the pre‐mRNA of DNMT3B was enriched in PTBP1 immunoprecipitated RNAs, compared with the negative control (Figure 4g,h). Correspondingly, PTBP1‐binding pyrimidine‐rich motifs (e.g., UCUU, UCUUC) were found widely on the upstream and downstream sequences of exon 5 in DNMT3B pre‐mRNA. To further identify the exact binding region for PTBP1 in DNMT3B pre‐mRNA, we synthesized 4 independent oligonucleotides (oligos) derived from different DNMT3B intron sequences. Strikingly, PTBP1 was shown to directly bind to Oligo 5‐1 according to RNA pull‐down assay (Figure 4i). However, the interaction between Oligo 5‐1 and PTBP1 could be disrupted by mutation of the PTBP1 binding site from UCUUC to ACAAC (Figure 4j). Given that PTBP1 contains four RNA recognition motifs (RRMs), we further identified the specific binding site with DNMT3B pre‐mRNA by constructing several deletion mutants of PTBP1 and confirmed their expression via WB assay (Figure 4k,l). Then, we transfected those constructs into DU145 cells and found that only RRM3 deletion failed to rescue DNMT3B exon 5 retention, indicating that RRM3 of PTBP1 is chiefly responsible for DNMT3B pre‐mRNA binding (Figure 4m,n). Collectively, these data demonstrate that PTBP1 regulates the AS of DNMT3B by directly binding to the motif located in intron 5, which is dependent mainly on the RRM3 domain.

### 2.5 PTBP1 Interacts with RALY to Regulate DNMT3B Splicing in PCa

**Figure 5:**
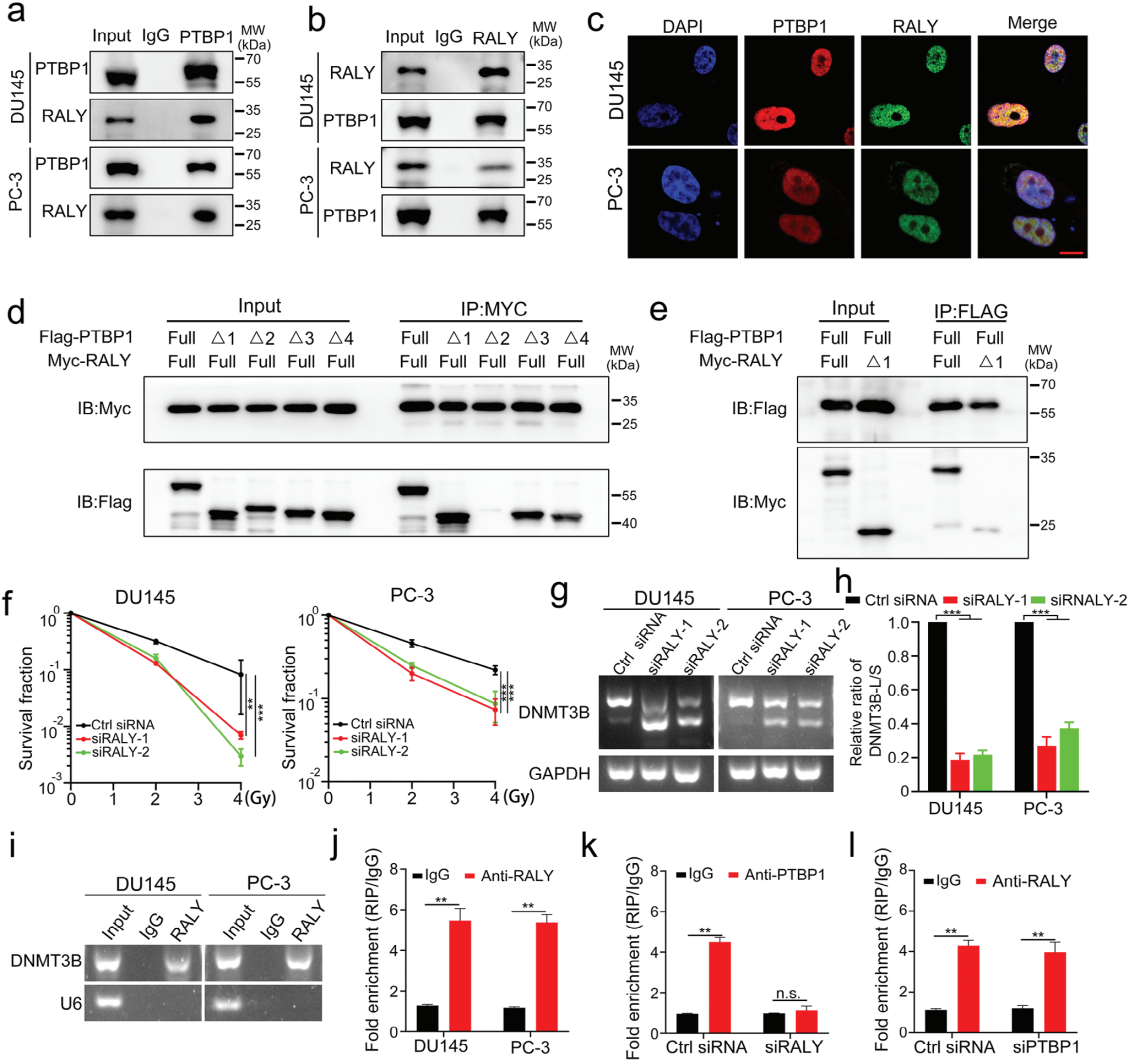
PTBP1 interacts with RALY to regulate DNMT3B splicing in prostate cancer cells a,b) Co‐immunoprecipitation analysis of the interaction between PTBP1 and RALY in prostate cancer (PCa) cells with an anti‐PTBP1 antibody (a) or anti‐RALY antibody (b). c) Immunofluorescence demonstrating the subcellular colocalization of PTBP1(red) and RALY (green) in PCa cells. Scale bar, 20 µm. d) Co‐immunoprecipitation analysis of the interaction between RALY and PTBP1 truncation variants with anti‐MYC antibody. e) Co‐immunoprecipitation analysis of the interaction between PTBP1 and RALY truncation variants with anti‐FLAG antibody. f) Clonogenic survival of PCa cells transiently transfected with the indicated siRNAs against RALY in response to irradiation (0, 2, and 4 Gy). g,h) Representative gel electrophoresis image (g) and statistical analysis (h) of the DNMT3B‐L/DNMT3B‐S ratio in RALY knockdown PCa cells by RT‐PCR. i,j) Representative images (i) and statistical analysis (j) of DNMT3B pre‐mRNA enrichment on RALY from RIP assay in PCa cells using anti‐RALY antibody. k) Statistical analysis of DNMT3B pre‐mRNA enrichment on PTBP1 from the RIP assay in RALY knockdown or control PCa cells using anti‐PTBP1 antibody. l) Statistical analysis of DNMT3B pre‐mRNA enrichment on RALY from the RIP assay in PTBP1 knockdown or control PCa cells using anti‐RALY antibody. The data are presented as the mean ± S.D.s of three independent experiments. *p < 0.05, **p < 0.01, ***p < 0.001 by two‐tailed Student's *t* test, or by two‐way ANOVA followed by Tukey's post‐hoc test where applicable. ns. no significance.

To gain deeper insight into the regulatory mechanism of PTBP1‐mediated AS, the proteins that interact with PTBP1 were identified in PCa cells via IP‐MS. A total of 308 identified proteins overlapped between DU145 and PC‐3 cells (Table S4, Supporting Information). We subsequently performed Gene Ontology analysis on the basis of the 308 shared proteins and spotted that the top‐ranked biological processes were RNA splicing and its associated regulatory pathways, reiterating the role of PTBP1 in PCa (Figure S4a, Supporting Information). Among the proteins, the heterogeneous nuclear ribonucleoproteins (hnRNP) RALY was the most enriched one with the highest protein sequence coverage (Figure S4b, Supporting Information). Co‐IP assay confirmed the specific interaction between PTBP1 and RALY (**Figure** **5**a,b). In parallel, co‐localization of PTBP1 and RALY was also detected in the nucleus of PCa cells via IF (Figure 5c). Serial deletion analysis revealed that RRM2 of PTBP1 and the C‐terminus of RALY were crucial for their integration (Figure 5d,e). Consistent with the role of PTBP1 in PCa, in vitro experiments showed that RALY KD prominently increased the radiosensitivity of PCa cells (Figure 5f; Figure S5, Supporting Information). Meanwhile, RALY KD also significantly attenuated the DNMT3B exon 5 inclusion, which was similar to the effect induced by PTBP1 KD, as observed by RT‐PCR (Figure 5g,h). Furthermore, RIP assay with RALY antibody revealed that DNMT3B pre‐mRNA was significantly enriched in the RALY immunoprecipitates compared with the negative control, indicating that RALY might interact with PTBP1 to regulate the AS of its target genes (Figure 5i,j). Next, to further examine whether RALY is required for PTBP1 binding to DNMT3B pre‐mRNA, we conducted a RIP assay with an anti‐PTBP1 antibody in RALY KD PCa cells. Intriguingly, the RT‐PCR results showed that the enrichment of DNMT3B pre‐mRNA in PTBP1‐containing immunoprecipitates was markedly attenuated in RALY KD PCa cells, compared with that in control PCa cells (Figure 5k). Conversely, PTBP1 KD did not abolish the interaction between RALY and the DNMT3B pre‐mRNA (Figure 5l). In addition, RALY KD significantly blocked PTBP1 OE‐mediated exon 5 inclusion of DNMT3B in PCa cells (Figure S6a,b, Supporting Information). Taken together, these results highlight the importance of RALY as a crucial contributor to PTBP1‐regulated AS of its targeted genes in PCa.

### 2.6 The Oncogenic Role of PTBP1 in PCa Relies Mainly on DNMT3B‐L Expression

**Figure 6:**
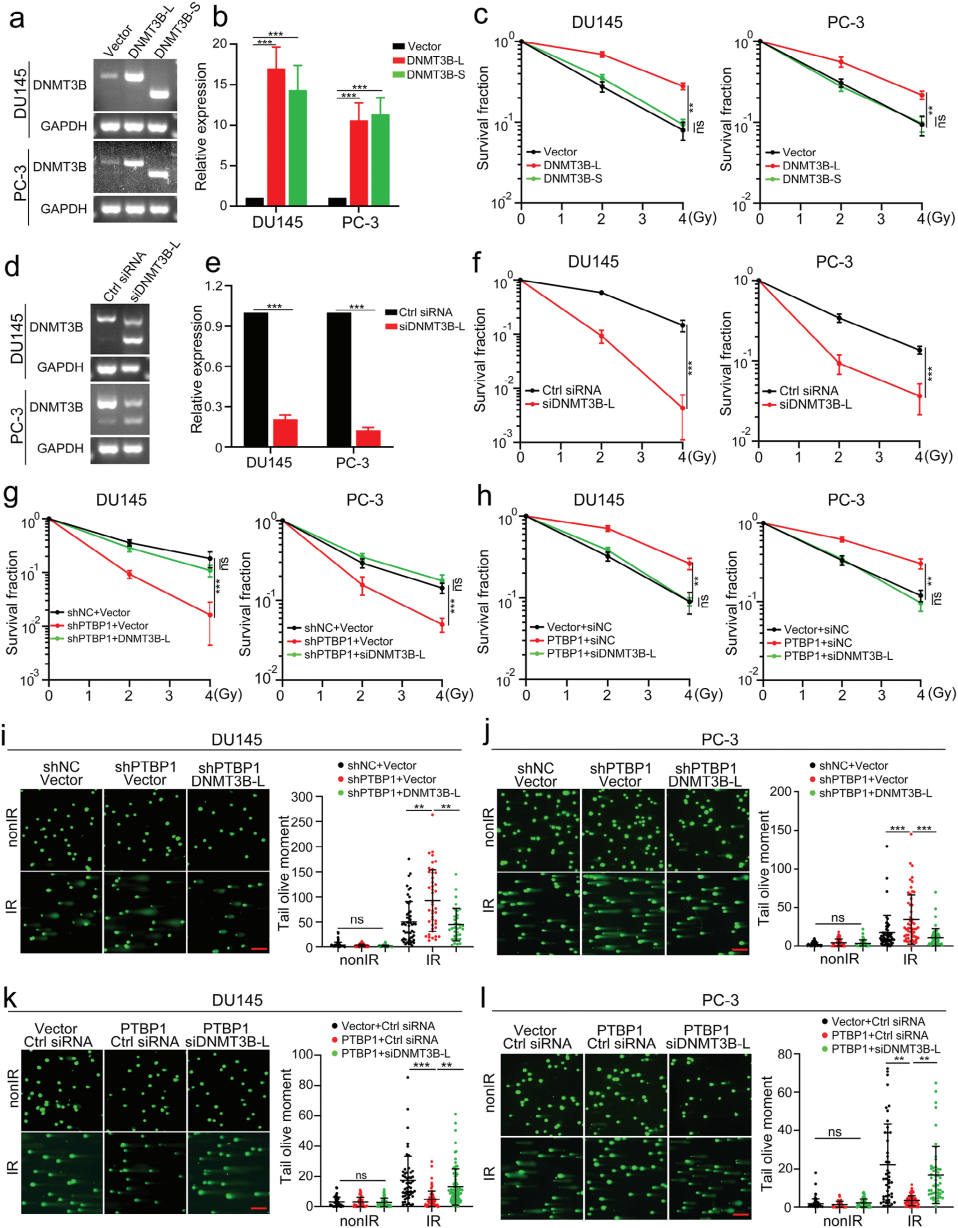
PTBP1 increases radioresistance of prostate cancer cells via a DNMT3B‐L‐dependent manner. a,b) Representative gel electrophoresis image (a) and statistical analysis (b) of the overexpression efficiency of DNMT3B‐L and DNMT3B‐S in prostate cancer (PCa) cells. c) Clonogenic survival in response to irradiation (0, 2, and 4 Gy) of PCa cells transfected with the indicated DNMT3B isoforms or control plasmids. d,e) Representative gel electrophoresis image (d) and statistical analysis (e) of the efficiency of DNMT3B‐L knockdown in PCa cells. f) Clonogenic survival in response to irradiation (0, 2, and 4 Gy) of PCa cells transfected with DNMT3B‐L siRNA or control siRNA. g,h) Clonogenic survival of PCa cells treated as indicated and exposed to irradiation (0, 2, and 4 Gy). i–l) Representative images (left) and statistical analyses (right) of the comet assay results of the indicated DU145 (i, k) and PC‐3 (j, l) cells at 24 h after 4 Gy irradiation. Scale bar, 20 µm. The data are presented as the mean ± S.D.s of three independent experiments. *p < 0.05, **p < 0.01, ***p < 0.001 by two‐tailed Student's *t* test, or by two‐way ANOVA followed by Tukey's post‐hoc test where applicable. ns. no significance.

To investigate the functional link between PTBP1 and DNMT3B in contributing to radioresistance in PCa, we first examined the expression of different DNMT3B isoforms in a tissue set comprising 14 pairs of PCa tissues via PCR and found that the percentage‐splice‐in (PSI) was significantly higher in PCa tissues than that in paired adjacent noncancerous tissues (Figure S7a, Supporting Information). Moreover, the DNMT3B‐L/S ratio was positively associated with PTBP1 expression in PCa tissues (Figure S7b, Supporting Information). Then, to further explore the functional roles of DNMT3B‐L and DNTM3B‐S in radioresistance, we performed gain‐of‐function assays (**Figure** **6**a,b). Strikingly, ectopic expression of DNMT3B‐L, but not DNMT3B‐S, resulted in increased radioresistance in vitro (Figure 6c; Figure S8a, Supporting Information). In contrast, DNMT3B‐L KD significantly diminished clonogenicity after IR, which confirmed that DNMT3B‐L was essential for inducing radioresistance in PCa (Figure 6d–f; Figure S8b, Supporting Information).

To determine whether DNMT3B‐L is an essential downstream target of PTBP1, rescue experiments were conducted. The results of the colony formation assay demonstrated that DNMT3B‐L OE rescued the survival of PTBP1‐deficient PCa cells almost back to control levels (Figure 6g; Figure S8c, Supporting Information). Meanwhile, DNMT3B‐L KD significantly abolished the PTBP1 OE‐induced increase in radioresistance in PCa cells (Figure 6h; Figure S8d, Supporting Information). Additionally, the results of the comet assay showed that PTBP1 KD‐induced DNA damage was effectively attenuated by DNMT3B‐L OE in IR‐treated PCa cells (Figure 6i,j). In contrast, DNMT3B‐L siRNA significantly enhanced IR‐induced DNA damage in PTBP1 OE PCa cells (Figure 6k,l). In summary, these results demonstrate that PTBP1 exerts its radioresistance‐enhancing effects in PCa cells via DNMT3B‐L.

### 2.7 PTBP1 Regulates Gene Expression via DNMT3B‐L‐Induced DNA Hypermethylation

**Figure 7:**
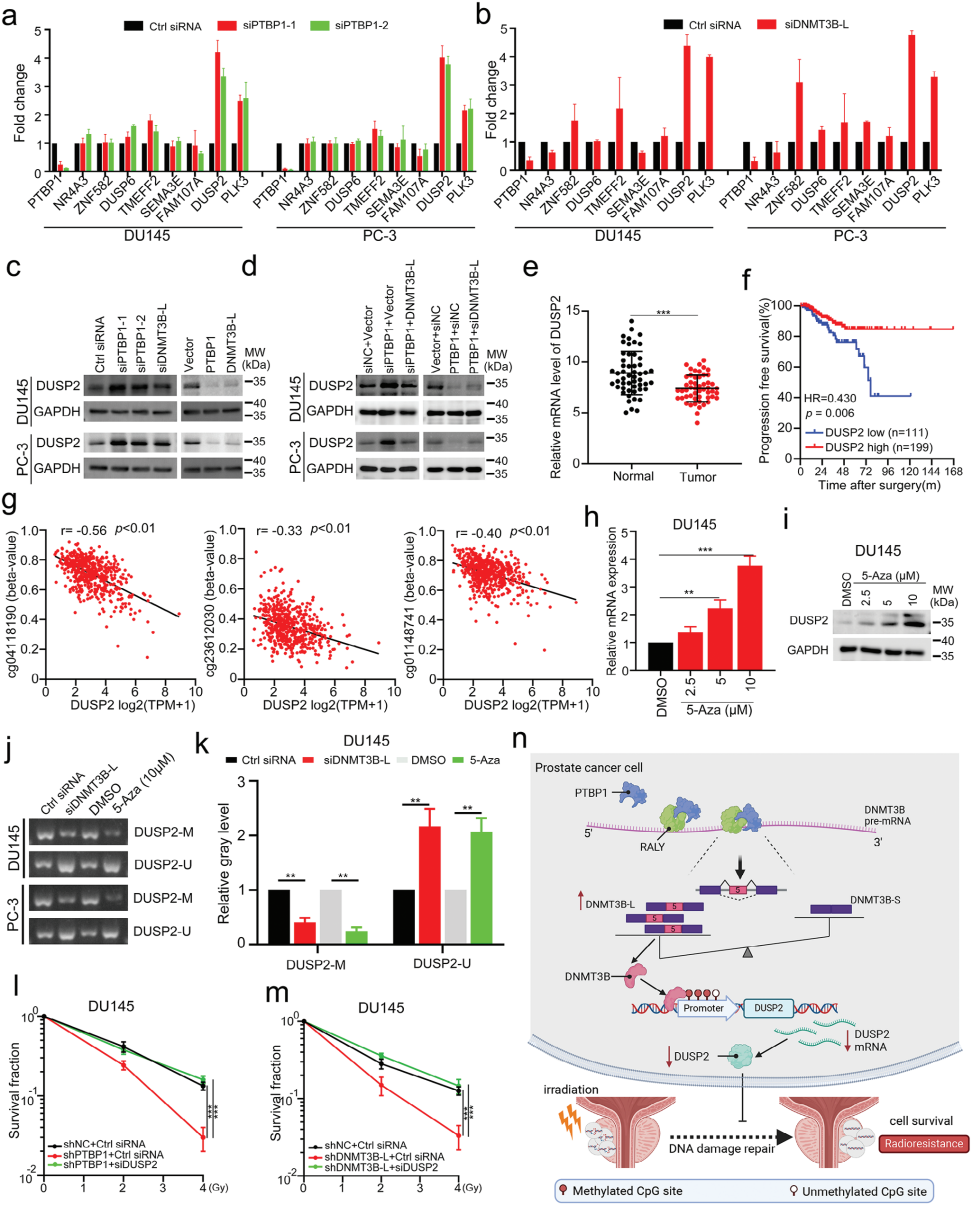
PTBP1 modulates DUSP2 through DNMT3B‐L mediated DNA methylation a,b) The expression of the indicated target genes was detected via qPCR in PTBP1(a) or DNMT3B‐L (b) knockdown prostate cancer (PCa) cells. c,d) The expression of DUSP2 was verified in PCa cells subjected to the indicated treatments via western blot assay. e) Difference in DUSP2 expression between PCa tissues and normal tissues in the TCGA database. f) Kaplan–Meier curves for progression‐free survival of PCa patients with high or low expression of DUSP2 in TCGA database. g) Correlation between DUSP2 expression and the methylation level of the indicated methylation sites in the TCGA data. h,i) DUSP2 expression in PCa cells treated with 5‐Aza was detected by qPCR (h) and western blotting (i). j,k) Representative gel electrophoresis image (j) and statistical analysis (k) of the methylation of DUSP2 promoter in PCa cells treated as indicated. l,m) Clonal survival of PCa cells subjected to the indicated treatment followed by irradiation. n) Schematic model of the mechanism underlying the role of PTBP1 in PCa radioresistance. The data are presented as the mean ± S.D.s of three independent experiments. *p < 0.05, **p < 0.01, ***p < 0.001 by two‐tailed Student's *t* test, or by two‐way ANOVA followed by Tukey's post‐hoc test where applicable. ns. no significance.

To explore how PTBP1 functions through regulating DNMT3B‐L, we performed RNA‐seq on PTBP1 or DNMT3B‐L KD and control PC‐3 cells and identified 843 genes with significant expression change (fold change >2.0). GO analysis revealed that both PTBP1 and DNMT3B‐L target genes were enriched in important cancer‐related pathways, including DNA damage respire (Figure S9, Supporting Information). Given that DNMT3B is a DNA methyltransferase that mainly inhibits target gene expression, we focused on genes whose expression concordantly increased in PTBP1 and DNMT3B‐L KD cells. Several known tumor suppressor genes were selected and validated via qRT‐PCR, with Dual‐Specificity Phosphatase‐2 (DUSP2) being one of the most distinctly upregulated genes (**Figure** **7**a,b). Consistent with these findings, WB analysis revealed that the expression of DUSP2 was consistently increased in PTBP1 or DNMT3B‐L KD PCa cells but consistently decreased in PTBP1 or DNMT3B‐L OE PCa cells (Figure 7c). Furthermore, DNMT3B‐L OE markedly blocked PTBP1 KD‐induced increases in the DUSP2 protein level (Figure 7d). In parallel, DNMT3B‐L KD rescued the attenuation of DUSP2 expression induced by PTBP1 OE, indicating that PTBP1 might reduce DUSP2 expression in a DNMT3B‐L‐dependent manner (Figure 7d). Additionally, we analyzed the TCGA data and found that DUSP2 expression was significantly downregulated in PCa tissues (Figure 7e). Low expression of DUSP2 was significantly correlated with poor progression‐free survival in patients with PCa (Figure 7f).

To further dissect the mechanism by which DNMT3B‐L regulates DUSP2, we first evaluated the CpG methylation status of the promoter and 5′‐upstream regions of DUSP2 on the basis of TCGA data. Interestingly, 3 of the 4 possible CpG sites upstream of DUSP2 were hypermethylated and negatively associated with DUSP2 expression in PCa (Figure 7g; Figure S10, Supporting Information). Moreover, in vitro experiments demonstrated that 5‐aza‐2′‐deoxycytidine (5‐Aza), a DNA methylation inhibitor, significantly increased the mRNA and protein levels of DUSP2 in a dose‐dependent manner, which further confirmed that DUSP2 expression could be regulated by DNA methylation (Figure 7h,i). Considering DNMT3B could facilitate CpG site methylation in the promoter region of target genes, we assumed that DNMT3B‐L might inhibit DUSP2 expression by increasing CpG site methylation in the upstream promoter region of DUSP2. To verify this hypothesis, we designed a pair of methylated and non‐methylated primers covering the above three CpG sites via online software (http://www.urogene.org/methprimer2/). Strikingly, the MSP results demonstrated that DNMT3B‐L KD significantly reduced the number of PCR products produced via the use of methylated primers but conversely increased the number of PCR products produced via the use of non‐methylated primers (Figure 7j,k). Similar effects were observed with 5‐Aza treatment (Figure 7j,k). To investigate whether DUSP2 displays a nonnegligible effect on both PTBP1 and DNMT3B‐L‐mediated radioresistance, we performed rescue experiments. Interestingly, DUSP2 siRNAs markedly restored the radioresistance of PTBP1 or DNMT3B‐L KD cells (Figure 7l,m; Figure S11a–d, Supporting Information). Meanwhile, DUSP2 KD significantly reduced IR‐caused DNA damage in PTBP1 or DNMT3B‐L KD cells (Figure S11e–h, Supporting Information). Collectively, these data demonstrate that PTBP1 facilitates radioresistance via DNMT3B‐L‐mediated promoter methylation of DUSP2 in PCa.

## 3 Discussion

Aberrant epigenetic modulation of cancer cells contributes to tumor progression and treatment resistance. Accumulating studies have indicated that the aberrant AS profile significantly increases the complexity of the oncogenic network and further facilitates cancer progression.^[^ ^24^ ^]^ However, the aberrant AS events and the corresponding splicing factors involved in the radioresistance of PCa remain largely unknown. In the present study, for the first time to our knowledge, we comprehensively analyzed differences in the expression of 374 known splicing factors between prostate cancer and adjacent noncancerous tissues via multiple public databases. We found that PTBP1 was obviously overexpressed in PCa tissues and significantly associated with advanced clinicopathological staging, which was further verified in two additional cohorts. In vitro and in vivo experiments demonstrated that PTBP1 enhanced PCa cell proliferation and survival under radiotherapy by expediting DNA damage repair. These findings highlight the nonnegligible contribution of splicing factors to the radioresistance of PCa.

Usually, RNA splicing is a highly complicated and orchestrated process accomplished by the spliceosome, along with regulatory splicing factor proteins. As one of the hnRNPs, PTBP1 mainly regulates AS by binding their specific cis‐acting sequences in pre‐mRNA, and subsequently influences tumor progress in various cancers. For example, Xu et al. found that PTBP1 drives liver cancer tumorigenesis by promoting cancer metabolic switch.^[^ ^25^ ^]^ Our previous data indicated that PTBP1 promotes proliferation and metastasis of bladder cancer.^[^ ^19^ ^]^ Differently from previous works, our data in this study first demonstrated that PTBP1 overexpression could efficiently elicit radiotherapy resistance of PCa cells by regulating aberrant exon skipping of DNMT3B, which further broadened knowledge about PTBP1 regulatory role in tumor progression. Well, with the comprehensive analysis of PTBP1‐related studies, we found that different functions and clinical relevance that PTBP1 may display among different cancers. One potential reason for these discrepancies is the distinct PTBP1‐governed AS networks among different tumor types. For instance, our data indicated that PTBP1 was not involved in the splicing regulation of MESI and PKM, which induce tumor proliferation in bladder cancer, indicating the context‐dependent nature of PTBP1 target selection. Notably, given that PTBP1 knockdown induces a similar percentage of exon exclusion and inclusion splicing events, we speculated that PTBP1 either activates or represses the splicing of the target exons. In addition, PTBP1 could facilitate liver cancer metastasis by activating the exclusion of Axl exon 10 to produce the Axl‐S protein isoform.^[^ ^26^ ^]^ Meanwhile, PTBP1 could also drive liver cancer glycolysis by activating the inclusion of PKM exon 10 to increase the PKM2 protein isoform.^[^ ^27^ ^]^ Thus, these data indicate that the differences in the manner of splicing may be mainly responsible for the functional diversity of PTBP1 in cancers.

DNA methylation, as a major epigenetic modification mechanism, regulates gene transcription involved in various physiological and pathophysiological processes.^[^ ^28^ ^]^ It was mainly catalyzed by three DNA methyltransferases (DNMT), DNMT1, DNMT3A, and DNMT3B.^[^ ^29^ ^]^ Among those DNMTs, DNMT3B is of interest because of its close links to cancer.^[^ ^30^ ^]^ It is reported that DNMT3B is aberrantly upregulated in several cancers and contributes to many aspects of tumor progression. Gang et al. detected that DNMT3B knockdown efficiently sensitized PCa cells to irradiation.^[^ ^31^ ^]^ Actually, more than 40 splice variants were identified for DNMT3B, but their functions and regulation remain largely unclear.^[^ ^32^ ^]^ Our data demonstrated that PTBP1 obviously repressed exon 5 skipping that compromises the expression of the truncated splicing variant DNMT3B through binding to the motif in the intron 5. Our experimental data also revealed that the full‐length DNMT3B variant containing exon 5 significantly enhanced the radioresistance of PCa cells, but the truncated DNMT3B variant without exon 5 failed to increase radioresistance in PCa. This may be attributed to the fact that catalytically inactive DNMT3B‐S exhibited enhanced DNA binding affinity and blocked access to other active isoforms of DNMT3B in a competitive binding manner, consequently leading to hypomethylation of targeted genes.^[^ ^30^ ^]^ Inconsistent with our data, a previous study showed that ectopic overexpression of DNMT3B‐S could significantly enhance colon cancer cell growth, indicating that DNMT3B‐S exerts its function depending on the cellular context.^[^ ^30^ ^]^ Additional experiments are required to explore the mechanism by which DNMT3B‐S influences tumor progression. Hence, our study revealed a new function and regulatory mechanism of DNMT3B splice isoforms in PCa.

Although splicing factors are important regulators for pre‐mRNA splicing, the sequences of splicing factor‐binding sites are conserved and can not fully explain aberrant splicing events in cancer cells. The splicing factor‐interacting factors that mediate the interaction between splicing factors and pre‐mRNAs may partly account for the aberrant cancer‐specific AS. Yang et al. detected that MTR4 could promote cancer metabolic reprogramming by recruiting PTBP1 to the pre‐mRNA of GLUT1.^[^ ^33^ ^]^ Yang et al. demonstrated that RALY activates the cholesterol synthesis pathway by facilitating SF3B3‐mediated AS of MAT1.^[^ ^34^ ^]^ In the present study, we confirmed that PTBP1 regulated AS of DNMT3B depending on RALY with the following evidence. First, Co‐IP experiments showed that PTBP1 interacted with RALY; Second, both PTBP1 and RALY could bind to the pre‐mRNA of DNMT3B and induced DNMT3B splicing switch from DNMT3B‐S to DNMT3B‐L in PCa cells; Third, RALY knockdown obviously reduced the binding abundance between PTBP1 and pre‐mRNA of DNMT3B. However, PTBP1 knockdown did not alter the interaction between RALY and pre‐mRNA of DNMT3B. Together with our present findings and previous studies, we found that one splicing factor contains different interacting proteins and splicing factors could share the same interacting protein in different cancers, which could potentially explain the discrepancies of AS events in various cancers.

DNMT3B‐mediated radioresistance was reported in various cancers, however, the mechanisms were heterogeneous.^[^ ^35^, ^36^ ^]^ The present study was the first to identify DUSP2 as a downstream gene of DNMT3B‐L and further demonstrated that DNMT3B‐L could increase the methylation of the DUSP2 promoter, thereby inhibiting DUSP2 expression. Importantly, DNMT3B‐L overexpression partly repressed the PTBP1 knockdown‐induced increase of DUSP2 expression. Moreover, DUSP2 knockdown obviously rescued the radioresistance of PCa cells with PTBP1 or DNMT3B‐L silence. Hence, we identified a novel mechanism that PTBP1 increased the radioresistance of PCa cells via DNMT3B‐L induced DUSP2 promoter methylation.

Although our data indicated that PTBP1‐mediated AS of DNMT3B enhanced radioresistance in PCa, further evaluation of PTBP1 expression in radioresistant patients is needed to confirm its clinical relevance. In addition, since DUSP2 was identified as a crucial mediator of PTBP1‐mediated radioresistance, more follow‐up experiments are required to illustrate the exact mechanism by which DUSP2 induced radioresistance to comprehensively elucidate the role of PTBP1 in radioresistance. Apart from DNMT3B, our RNA‐seq data demonstrated that PTBP1 also induced AS of other genes, thus, further studies are warranted to explore whether the AS of these candidate genes similarly contributes to radioresistance in PCa or influences another process, such as metastasis.

In summary, we identified PTBP1 as an oncoprotein by systematically analyzing the splicing factors in PCa. With the assistance of RALY, PTBP1 can bind to the pre‐mRNA of DNMT3B and drive an oncogenic splicing switch in DNMT3B to produce DNMT3B‐L, which in turn induces promoter methylation of DUSP2, thereby increasing resistance of cancer cells to irradiation (Figure 7n). Our study provides a novel insight into the mechanism, by which aberrant splicing factor regulates PCa radioresistance, and identifies PTBP1 as a potential therapeutic target for sensitization of PCa to radiotherapy.

## 4 Experimental Section

### Clinical Samples

After approval was obtained from the Internal Review and Ethics Board of Sun Yat‐sen Memorial Hospital (SYMH, approval number: SYSEC‐KY‐KS‐2020‐201) and Sun Yat‐sen University Cancer Center (SYSUCC, approval number: 2021‐FXY‐139), a total of 126 formalin‐fixed, paraffin‐embedded (FFPE) PCa samples from SYSUCC between 2005 and 2014 (named cohort 1), and another 113 FFPE PCa samples from SYMH between 2009 and 2013 (named cohort 2) were collected with informed written consents.^[^ ^37^ ^]^ All samples were reconfirmed by two senior pathologists through H&E staining for pathological identification. The detailed clinicopathological features of the 239 patients in both cohorts are listed in Table S1 (Supporting Information).

### Cell Lines, Antibodies, Plasmids, and Reagents

The cell lines (DU145, PC‐3, and HEK293T) utilized in this study were procured from the ATCC. DU145 and HEK293T cells were cultured in DMEM, and PC‐3 cells were cultured in RPMI 1640 supplemented with 10% FBS and 1% penicillin/streptomycin. All the cells were cultured at 37 °C with a CO_2_ concentration of 5%. Regular testing via the EEZ‐PCR mycoplasma test kit (BI) was conducted to ensure the absence of mycoplasma contamination. The antibodies, plasmids, and reagents used in the present study are listed in Table S5 (Supporting Information).

### Immunohistochemistry (IHC) and H‐Scores

IHC assay was conducted to measure the protein levels of candidate genes in human PCa tissues and mouse xenograft tissues via the indicated antibodies following standard procedures.^[^ ^38^ ^]^ The staining score of each slice was determined by multiplying the product of the staining intensity score (0–3) and the percentage of cell‐stained cells (0–100%). The cut‐off value of the H‐score for PTBP1 was set at 95 according to the X‐tile program.

### Transient Transfection and Construction of Stable Cell Lines

siRNAs synthesized by Gene Pharma were transiently transfected into PCa cells via Lipofectamine RNAiMAX (Invitrogen), and the target sequence of each siRNA is listed in Table S6 (Supporting Information). Transfection with plasmids was performed via Lipofectamine 2000 (Invitrogen) according to the manufacturer's introductions. For lentivirus generation, the pLKO.1‐shPTBP1‐1, pLKO.1‐shPTBP1‐2, pLKO.1‐shDNMT3B‐L, pCDH‐PTBP1, pCDH‐DNMT3B‐L or vectors were transfected into HEK293T cells along with the packaging plasmid psPAX2 and envelope plasmid pMD2.G, following the previous study.^[^ ^39^ ^]^ Next, DU145 and PC‐3 cells were infected with the lentivirus for 72 h and subsequently selected with 2 µg mL^−1^ puromycin for 2 weeks. Real‐time PCR and/or western blot assays were conducted to verify the knockdown or overexpression efficiency.

### Cell Proliferation and X‐ray Radiation Assays

CCK‐8 and colony formation assays were used to evaluate cell viability. For the colony formation assay, following seeding in 6‐well plates, the cells were exposed to varying doses of X‐ray radiation (0, 2, 4 Gy) and subsequently cultured for 2 weeks. Then, the cells were fixed with methanol and stained with crystal violet. Survival curves were calculated on the basis of the single‐hit multitarget model: SF = 1 − (1 − e (^−kD^)) ^N^.^[^ ^35^ ^]^ Ethynyl deoxyuridine (EdU) assays were performed to detect the proportion of proliferating cells via an EdU assay kit (RiboBio).^[^ ^40^ ^]^ Each experiment was repeated at least three times independently.

### Apoptosis Assay

Apoptosis assay was conducted using an Annexin V‐FITC/PI Apoptosis Detection Kit (KeyGen). Briefly, the treated cells were digested and resuspended in PBS. Then, the cells were sequentially incubated with Annexin V‐FITC antibody and propidium iodide. Finally, apoptosis was analyzed using CytExpert (version 24).^[^ ^40^ ^]^ Each experiment was repeated at least three times independently.

### Alkaline Comet Assay

After exposure to 4 Gy radiation, the degree of DNA damage to PCa cells was measured using a comet assay kit (R&D, USA) according to the manufacturer's instructions and the previous study.^[^ ^40^ ^]^ Briefly, treated PCa cells were suspended in low‐melting agarose, and subsequently transferred onto comet slides. Next, the comet slides were immersed in a specific lysis buffer and then alkaline solution (pH > 13), followed by electrophoresis at 18 volts cm^−1^. The Olive tail moment, which reflects the extent of DNA damage, was measured using an Olympus IX51 microscope and calculated using Cometscore 2.0 software. Each experiment was repeated at least three times independently.

### RNA Isolation, qRT‐PCR, and RNA‐seq

Total RNA extraction from TRIzol reagent (Invitrogen) and reverse transcribed to cDNA utilizing a cDNA reverse transcription kit (Takara Bio) following standard procedures. Quantitative real‐time PCR was exerted via the SYBR Green PCR kit (Takara Bio) with specific primers. GAPDH was used to normalize the target gene expression. For RNA‐seq, the extracted total RNA was used for cDNA library generation. The sequencing was subsequently conducted on an Illumina platform (Annoroad, Beijing, China). AS events were quantified by rMATS. The raw RNA‐sequencing data have been uploaded to the Gene Expression Omnibus (GSE276977). All primer sequences are included in Table S7 (Supporting Information).

### Western Blotting (WB), Co‐Immunoprecipitation (Co‐IP), and Mass Spectrometry (MS)

The WB assay was performed following the standard procedure as previously described.^[^ ^41^ ^]^ The primary antibodies specific for PTBP1, Ki67, γ‐H2AX, RALY, Flag, Myc, and GAPDH used are described in Table S2 (Supporting Information). For Co‐IP, cell lysates were immunoprecipitated with appropriate antibodies and then incubated with magnetic protein A/G beads. Finally, the bound proteins were boiled and subjected to WB analysis. Additionally, after washing with elution buffer, the immobilized immune complexes were subjected to proteomics screening by mass spectrometry on a MALDI‐TOF MS instrument (Bruker Daltonics).

### Immunofluorescence Staining (IF)

The IF assay was performed as described in the previous study.^[^ ^42^ ^]^ PCa cells were fixed in 4% paraformaldehyde, and permeabilized with 0.1% Triton X‐100. After that, the cells were incubated overnight with the indicated primary antibodies, followed by incubation with fluorescent secondary antibodies the next day. Finally, the cells were counterstained with DAPI and imaged via a confocal laser scanning microscope (Olympus, Japan).

### Tumor Xenograft Models

All animal experiments in the present study were approved by the Institutional Animal Care and Use Committee of Sun Yat‐sen University (approval number L102042019070B). Four‐week‐old male BALB/c nude mice were purchased from Shanghai SLAC Laboratory Animal Co., Ltd., and housed under specific pathogen‐free conditions. For radiation treatment, 2 × 10^6 ^PC‐3 cells transfected with vector or PTBP1 were suspended in PBS containing Matrigel and injected into the right flanks of nude mice. The tumor volume was measured with calipers and calculated according to the following formula: 0.5 × length × width.^2^ When the tumor volume reached approximately 100mm,^3^ the xenograft‐bearing nude mice were randomly divided into two groups to receive mock IR or 4 Gy IR twice at 1‐week intervals. Tumor volumes were measured every 3 days. The mice were euthanized at the same time when tumors in any group reached ≈1400 mm^3^.^[^ ^43^ ^]^

### RNA Immunoprecipitation (RIP)

RIP was carried out with the Magna RIP^TM^ RNA‐Binding Protein Immunoprecipitation Kit (Millipore, Bedford, MA). Briefly, PCa cells were collected and lysed in RIP lysis buffer containing protease inhibitor and RNase inhibitor. Then, the cell lysates were incubated overnight at 4 °C with PTBP1, RALY, Flag, or Myc antibody‐bound A/G beads or control IgG‐conjugated A/G beads. Finally, the co‐precipitated RNAs were extracted via the RNeasy MinElute Cleanup Kit (Qiagen) and subjected to RT‐PCR analysis.^[^ ^44^ ^]^

### RNA Pull‐Down Assays

Bio‐labeled probes were synthesized by GenePharma (Shanghai, China), and the probe sequences are shown in Table S8 (Supporting Information). The RNA pull‐down assay was carried out via a magnetic RNA‐protein pull‐down kit (Thermo Scientific, USA), as described in the previous study.^[^ ^45^ ^]^ Briefly, the probes were first incubated with streptavidin magnetic beads for 30 min. Then, PCa cell lysates were added to each binding interaction and incubated for 1 h. Following washing with IP lysis buffer, the retrieved proteins were ultimately isolated for WB assay.

### RNA Fluorescence In Situ Hybridization (RNA‐FISH)

RNA‐FISH was conducted as described previously.^[^ ^45^ ^]^ Cy3‐labeled DNMT3B‐L and FAM‐labeled DNMT3B‐S probes were synthesized by GenePharma (Shanghai, China), and the probe sequences are listed in Table S9 (Supporting Information). In brief, after transfection with PTBP1 siRNA or negative control for 48 h, PCa cells were fixed with 4% paraformaldehyde and subsequently permeabilized with 0.5% Triton X‐100. Then, following prehybridization, PCa cells were hybridized with the probe (5 mM) overnight. Finally, the nuclei were stained with 4,6‐diamidino‐2‐phenylindole (DAPI). Images were captured using a confocal microscope (Zeiss, Munich, Germany).

### Methylation‐Specific PCR (MSP) Analysis

The genomic DNA of PCa cells was extracted via the DNeasy Tissue Kit (Qiagen). Then, the EZ DNA methylation‐gold^TM^ Kit (Qiagen) was used for bisulfite conversion, which converts unmethylated cytosines, but not methylated cytosines, to uracil. PCR was performed to identify the unmethylated and methylated DUSP2 sequences.^[^ ^46^ ^]^ The primers used for MSP are provided in Table S4 (Supporting Information).

### Statistical Analysis

GraphPad Prism 9 and SPSS version 24.0 were carried out to generate graphs and perform the statistical analysis. The quantitative data were harvested from at least three independent experiments and presented as mean ± standard deviations (S.D.s). Student's *t*‐test for two groups or one‐way ANOVA for more than two groups were used to determine significant differences. Post‐hoc analyses were applied to control for multiple comparisons where appropriate. Pearson's chi‐square test was performed to assess clinical variables. Pearson's correlation coefficient was calculated to determine the correlation between two quantitative variables. Survival curves were generated using the Kaplan–Meier method and statistically calculated by the log‐rank test. A Cox proportional hazards model was used to estimate the adjusted survival hazard ratios (HRs) and 95% confidence intervals (CIs). *p* < 0.05 was considered statistically significant.

### Ethics Approval and Consent to Participate

This study was approved by the Institutional Ethical Boards of SYSMH (SYSEC‐KY‐KS‐2020‐201) and SYSUCC (2021‐FXY‐139), and appropriate informed consent was obtained from each patient.

## Conflict of Interest

The authors declare no conflict of interest.

## Author Contributions

H.X.H., Q.H.Z., Y.J.Z., and Y.L. contributed equally to this work. X.C., S.M.B., and T.X.L. conceived and designed the research; H.X.H., Q.H.Z., and Y.J.Z. performed the cell line experiments in vitro; H.X.H., Y.J.Z., and Y.L. performed the xenograft tumor experiments; L.D., T.S., S.L., SM.P., and H.X.H. collected the clinicopathological data; J.Y., L.T.L., M.H., and L.C. completed the follow‐up; Q.Z., H.Z., and J.L.L. contributed to the computational analysis of the gene signature and the statistical analysis; RH.X. contributed reagents; H.X.H., Q.H.Z., and X.C. wrote the manuscript; all authors reviewed and approved the manuscript for publication.

## Supporting information



Supporting Information

## Data Availability

The data that support the findings of this study are available from the corresponding author upon reasonable request.
