# Supplementary material for: PTBP1 Regulates DNMT3B Alternative Splicing by Interacting With RALY to Enhance the Radioresistance of Prostate Cancer
Source: Adv Sci (Weinh). 2024 Sep 17;11(42):2405997. doi: 10.1002/advs.202405997 (PMC11558147; doi:10.1002/advs.202405997)
Supplement: Supplementary file 1 — Supporting Information [file ADVS-11-2405997-s001.docx]

**Supplemental materials**

**Table S1.** Basic characteristics of prostate cancer patients in two cohorts

| Variables |  | Cohort 1 (n=126) |  | Cohort 2(n=113) |
| --- | --- | --- | --- | --- |
|  |  | Number of cases (%) |  | Number of cases (%) |
| Age (y, Mean range) | 66(42-89)  <66  ≥66 | 59(46.8%)  67(53.2%) |  | 41(36.3%)  72(63.7%) |
| T stage | T2  T3  T4 | 76(60.3%)  30(23.8%)  20(15.9%) |  | 80(70.8%)  28(24.8%)  5(4.4%) |
| N stage | N0  N1 | 116(92.1%)  10(7.9%) |  | 110(97.3%)  3(2.7%) |
| M stage | M0  M1 | 116(92.1%)  10(7.9%) |  | 105(92.9%)  8(7.1%) |
| Gleason score | 10  9  8  7(4+3)  7(3+4)  ≤6 | 4(3.2%)  23(18.2%)  16(12.7%)  18(14.3%)  24(19.0%)  41(32.6%) |  | 3(2.7%)  11(9.7%)  9(8.0%)  36(31.9%)  28(24.8%)  26(23.0%) |
| PTBP1 | Low  High | 55(43.7%)  71(56.3%) |  | 63(55.8%)  50(44.2%) |

**Table S2**. Univariate analysis of prognostic factors correlated with OS and CSS

| Variables | OS | | | | |  | CSS | | | | |
| --- | --- | --- | --- | --- | --- | --- | --- | --- | --- | --- | --- |
|  | Cohort 1 | |  | Cohort 2 | |  | Cohort 1 | |  | Cohort 2 | |
|  | HR  (95%CI) | *p*-value |  | HR  (95%CI) | *p*-value |  | HR  (95%CI) | *p*-value |  | HR  (95%CI) | *p*-  value |
| Age (y)  ≥66/<66 | 1.94  0.98-3.88 | 0.059 |  | 1.42  0.60-3.40 | 0.423 |  | 1.89  0.83-4.32 | 0.129 |  | 1.21  0.43-3.41 | 0.713 |
| Gleason score 7(4+3)-10/6-7(3+4) | 2.58  1.27-5.24 | **0.009** |  | 7.28  2.14-24.67 | **0.001** |  | 2.46  1.06-5.70 | **0.036** |  | 7.06  1.59-31.39 | **0.010** |
| Tumor stage  T3-4/T2 | 2.14  1.10-4.17 | **0.025** |  | 5.50  2.30-13.16 | **<0.001** |  | 2.30  1.03-5.13 | **0.042** |  | 6.03  2.06-17.71 | **0.001** |
| Distal metastasis  Present/Absent | 5.83  2.63-12.93 | **<0.001** |  | 4.43  1.63-12.03 | **0.003** |  | 6.49  2.56-16.43 | **<0.001** |  | 7.27  2.48-21.30 | **<0.001** |
| PTBP1  High/Low | 3.25  1.48-7.15 | **0.003** |  | 4.26  1.73-11.35 | **0.002** |  | 2.39  1.08-5.30 | **0.031** |  | 6.35  1.79-22.60 | **0.004** |

Abbreviations: OS, overall survival; CSS, cancer-specific survival; HR hazard ratio, CI confidence interval; p-value<0.05 marked in bold font shows statistically significant.

**Table S3**. Multivariate analysis of prognostic factors correlated with OS and CSS

| Variables | OS | | | | |  | CSS | | | | |
| --- | --- | --- | --- | --- | --- | --- | --- | --- | --- | --- | --- |
|  | Cohort 1 | |  | Cohort 2 | |  | Cohort 1 | |  | Cohort 2 | |
|  | HR  (95%CI) | *p*-value |  | HR  (95%CI) | *p*-value |  | HR  (95%CI) | *p*-value |  | HR  (95%CI) | *p*-  value |
| Gleason score 7(4+3)-10/6-7(3+4) | 1.25  0.52-3.03 | 0.620 |  | 3.79  1.03-13.93 | **0.045** |  | 1.05  0.37-2.97 | 0.929 |  | 2.78  0.56-13.93 | 0.213 |
| Tumor stage  T3-4/T2 | 1.58  0.79-3.16 | 0.198 |  | 3.08  1.18-8.03 | **0.022** |  | 1.67  0.73-3.90 | 0.223 |  | 2.65  0.77-9.09 | 0.121 |
| Distal metastasis  Present/Absent | 5.10  2.25-11.59 | **<0.001** |  | 2.023  0.67-6.13 | 0.213 |  | 5.84  2.23-15.30 | **<0.001** |  | 3.70  1.08-12.68 | **0.037** |
| PTBP1  High/Low | 2.72  1.04-7.09 | **0.041** |  | 2.73  0.99-7.55 | 0.053 |  | 3.47  1.06-11.34 | **0.040** |  | 4.37  1.14-16.79 | **0.032** |

Abbreviations: OS, overall survival; CSS, cancer-specific survival; HR hazard ratio, CI confidence interval; p-value<0.05 marked in bold font shows statistically significant.

**Table S4.** The binding proteins of PTBP1 are listed as follows.

| Gene names | | | | | |
| --- | --- | --- | --- | --- | --- |
| ABCF1 | CSE1L | FLNC | LRRC4B | PARP12 | RMDN1 |
| ABCF2 | CUX2 | FOCAD | LRRC59 | PARP2 | RNF213 |
| ACTR2 | DARS1 | FOXF2 | LRRC66 | PCARE | ROCK2 |
| ACTR3 | DBF4B | FUBP3 | LUC7L2 | PCBP2 | RPGRIP1L |
| ADAR | DDX19B | GARS1 | MAGED1 | PCSK2 | RPL12 |
| ADGB | DDX21 | GFPT1 | MAGED2 | PDZD2 | RPL13A |
| AFG3L2 | DDX28 | GNL3 | MAGIX | PEG10 | RPL26L1 |
| AFTPH | DDX6 | GPAT3 | MAGT1 | PFN1 | RPL36 |
| AGO2 | DHX15 | H1-0 | MAP10 | PGK1 | RPL5 |
| AIMP1 | DHX30 | H1-10 | MAP1B | PGM2 | RPL9 |
| ANK3 | DHX32 | H2BC21 | MAP2K1 | PGP | RPS24 |
| ANXA2 | DHX36 | HADHB | MARF1 | PHB | RPS26P11 |
| AP2A1 | DIAPH2 | HDAC1 | MASP1 | PHF6 | RPS8 |
| AP2B1 | DNAH10 | HDGFL2 | MBNL1 | PIK3CA | RPUSD3 |
| AP2M1 | DUSP13 | HELZ2 | MDH2 | PKP3 | RRBP1 |
| AP3B1 | E2F2 | HJURP | MKRN1 | PLEKHH2 | RREB1 |
| AP3M1 | EFTUD2 | HNRNPA0 | MLH1 | PLS3 | RSL1D1 |
| APOBEC3C | EHD1 | HNRNPA1L2 | MRPL15 | POLR2B | RUFY1 |
| APOBEC3G | EIF2AK2 | HNRNPL | MRPL18 | POLR2H | SCAMP3 |
| ARFGEF1 | EIF3A | HSPA9 | MRPL2 | POLR3A | SDE2 |
| ARHGEF18 | EIF3F | HYOU1 | MRPL43 | PPME1 | SETBP1 |
| ATM | EIF3I | IARS1 | MRPS18A | PPP2R1A | SF3B3 |
| ATP6V1A | EIF3L | IL17RC | MRPS22 | PRDX6 | SLC16A1 |
| ATXN2L | EIF4A3 | ILF3 | MRPS34 | PROSER3 | SLC22A23 |
| BAZ2A | EIF4E | IMMT | MRPS9 | PRPF8 | SLC25A10 |
| BTAF1 | EIF4G1 | ITGA6 | MSI2 | PRPS1 | SLC35C1 |
| BZW1 | EIF4G2 | KARS1 | MYBBP1A | PRSS1 | SLC39A13 |
| C16orf96 | EMD | KAT2A | MYL9 | PSMD3 | SMC2 |
| C3 | EPHA2 | KCNJ16 | MYRF | PTBP3 | SMG5 |
| C5 | EPS15L1 | KHDRBS3 | NDUFAF8 | PTPRF | SMG7 |
| CAPZB | ESCO2 | KPNA2 | NEK4 | PUM1 | SNRNP200 |
| CCAR2 | ESRP2 | KPNA5 | NEMF | PUM2 | SNRNP70 |
| CCT2 | ETAA1 | KRT35 | NFX1 | QKI | SNRPA1 |
| CCT5 | ETFB | L1RE1 | NOLC1 | RALY | SNRPD3 |
| CCT8 | EXOSC2 | LAMA5 | NONO | RANBP1 | SNX27 |
| CDC42BPB | FADS2 | LAMB3 | NOP58 | RAP1B | SORBS3 |
| CDK9 | FAM120C | LARS1 | NPEPPS | RARS1 | SPATS2 |
| COPA | FAM83H | LCLAT1 | NPM1 | RASGEF1C | SPATS2L |
| COPE | FAM98B | LDHB | OCLN | RBM25 | SPCS2 |
| COPG1 | FAT2 | LEMD1 | OR10K2 | RBM45 | SRP14 |
| CPOX | FBL | LINC00472 | PAICS | RBM7 | SRP68 |
| CRACD | FEN1 | LPCAT1 | PALMD | RBPMS | SRRM1 |
| CSDE1 | FERMT3 | LRIG1 | PAPLN | RDH11 | SRRM2 |
| YTHDC1 | ZC2HC1A | ZC3HAV1 | ZMYM4 | ZNF507 | ZNF521 |
| SRSF1 | SRSF10 | SRSF3 | SSB | SSBP1 | STAU2 |
| STT3A | STT3B | STX16 | SUGP2 | SYNM | TARBP2 |
| TARDBP | TARS1 | TASOR2 | TBC1D10A | TBRG4 | TDRD15 |
| TEKT2 | THBS2 | THEMIS | TLN2 | TMPO | TMX1 |
| TNPO1 | TNRC6A | TOP2A | TOR1A | TPI1 | TRA2B |
| TRIM28 | TRIM56 | TRPV5 | TTLL9 | TUT4 | TXNRD2 |
| U2AF2 | UBASH3B | UNC45B | WDR1 | WRAP53 | WTIP |
| WWC2 | ZNF638 |  |  |  |  |

**Table S5.** The antibodies used in this study are listed as follows.

| **Reagent or Resource** | **Source** | **Identifier** |
| --- | --- | --- |
| **Antibodies** |  |  |
| Rabbit anti-PTBP1 | CST | Cat#72669 |
| Rabbit anti-PTBP1 | Abcam | Cat#ab133734 |
| Rabbit anti-ki67 | CST | Cat#34330 |
| Rabbit anti-γ-H2AX | Abcam | Cat#ab229914 |
| Rabbit anti-RALY | Abcam | Cat#ab170105 |
| Anti-Flag | CST | Cat#14793 |
| Anti-Myc | CST | Cat#2276 |
| Anti-GAPDH | CST | Cat#5174 |
| Normal rabbit IgG | CST | Cat#2729 |
| Rabbit IgG (H+L) | CST | Cat#14708 |
| Mouse IgG (H+L) | CST | Cat#14709 |
| **Chemicals** | | |
| Decitabine | Selleck | Cat#S1200 |
| Lipofectamine RNAiMAX | Invitrogen | Cat# 13778100 |
| Lipofectamine 2000 | Invitrogen | Cat# 11668500 |
| Polybrene | Selleck | Cat# E1299 |
| **Recombinant DNA** |  |  |
| pCDH-3×Flag-PTBP1 | This paper | N/A |
| pCDNA3-3×Flag-PTBP1 | Song’s lab | N/A |
| pCDNA3-3×Flag-PTBP1-△RRM1 | Song’s lab | N/A |
| pCDNA3-3×Flag-PTBP1-△RRM2 | Song’s lab | N/A |
| pCDNA3-3×Flag-PTBP1-△RRM3 | Song’s lab | N/A |
| pCDNA3-3×Flag-PTBP1-△RRM4 | Song’s lab | N/A |
| pCDNA3-3×Myc-RALY | This paper | N/A |
| pCDNA3-3×Flag-RALY-△RRM | This paper | N/A |
| pCDNA3-3×Flag-PTBP1 | This paper | N/A |
| pCDNA3-DNMT3B-L | This paper | N/A |
| pCDNA3-DNMT3B-S | This paper | N/A |
| pMD2G | This paper | N/A |
| psPAX2 | This paper | N/A |
| pLKO.1-shPTBP1-1 | This paper | N/A |
| pLKO.1-shPTBP1-2 | This paper | N/A |
| pLKO.1-shDNMT3B-L | This paper | N/A |
| pLKO.1-Scramble | This paper | N/A |
| **Critical Commercial Assays** | | |
| Pierce Silver Stain for Mass Spectrometry Kit | Thermo Scientific | Cat#24600 |
| Pierce Crosslink Magnetic Co-IP kit | Thermo Scientific | Cat#26147 |
| Magnetic RNA-protein pull-down kit | Thermo Scientific | Cat#20164 |
| Magna RIP^TM^ RNA-Binding Protein Immunoprecipitation Kit | Millipore | Cat#17-701 |
| EZ DNA methylation-gold^TM^ Kit | Qiagen | Cat#D5030 |

**Table S6.** Sequences of siRNA oligos and shRNAs are listed as follows.

| **Name** | **Sequence 5’-3’** |
| --- | --- |
| **siRNA** |  |
| Si-Ctrl | UUCUCCGAACGUGUCACGUTT |
| Si-PTBP1-1 | CCCUCAUUGACCUGCACAATT |
| Si-PTBP1-2 | GCACAGUGUUGAAGAUCAUTT |
| Si-DNMT3B-L | CATATACAGTGGCTACATCTT |
| Si-RALY-1 | CGACTTCTACGACAGGCTCTT |
| Si-RALY-2 | TGTGTCTGTCTGTGAGCCTTT |
| Si-DUSP2 | CGAACUCGAAAUAACAACATT |
| **shRNA** |  |
| Sh-Ctrl | CAACAAGATGAAGAGCACCAA |
| Sh-PTBP1-1 | GCACAGTGTTGAAGATCATCA |

**Table S7.** Primers used in the present study

| **Name** | **Sequence 5’-3’** |
| --- | --- |
| **Used in MSP** |  |
| DUSP2-M Forwad | GGTGGGGTATTTTAAGGGTTATC |
| DUSP2-M Reverse | AAAAAAATATTACCCGAAATCGAA |
| DUSP2-U Forwad | GTGGGGTATTTTAAGGGTTATTGT |
| DUSP2-U Reverse | AAAAAAAATATTACCCAAAATCAAA |
| **Used in qPCR** |  |
| GAPDH Forward | GTCTCCTCTGACTTCAACAGCG |
| GAPDH Reverse | ACCACCCTGTTGCTGTAGCCAA |
| PTBP1 Forward | AATGACAAGAGCCGTGACTAC |
| PTBP1 Reverse | GGAAACCAGCTCCTGCATAC |
| DNMT3B-L Forward | ATGGCTCTGACACCCCAGT |
| DNMT3B-L Reverse | GACTCGTCCACATGGTTGC |
| DNMT3B-S Forward | AGCCCAGCTTCCCTGAGAC |
| DNMT3B-S Reverse | GTCTCCATCTCCACTGTCTGC |
| RALY Forward | GCAAGAAGAAGGGTGATGGA |
| RALY Reverse | TCTTCCTCGCTGTGTGTCAG |
| NR4A3 | CAAACCAAAGAGCCCATTACA |
| NR4A3 | TAACATGGTGCCAGACACAAA |
| ZNF582 Forward | GGCTTTTAGTCATTGCTCACAA |
| ZNF582 Reverse | TAAGGGGTAACTGCTGATGGA |
| DUSP6 Forward | CTGTCGATGAACGATGCCTAT |
| DUSP6 Reverse | TGGGGTCTTTCACGTAGATTG |
| SEMA3E Forward | TTACTGTGCTTGGGATGGTTC |
| SEMA3E Reverse | TCTTCATTTCGCCTCTGGAAT |
| TMEFF2 Forward | AGAAAGTGCCAGAGAACACCA |
| TMEFF2 Reverse | CACCACACAGATGACAGCAAT |
| FAM107A Forward | GAAGAAGAAGGAGGAGCTGGA |
| FAM107A Reverse | CTGGTCAGTGTGGCAATTCTC |
| DUSP2 Forward | CTTGCAGACTGGGTGAAGAAC |
| DUSP2 Reverse | ACTGAAGTTGGGGGAGATGAC |
| PLK3 Forward | TGCTGTTTAGTGATGGCACTG |
| PLK3 Reverse | AGGTGGGAAGCGAGGTAAGTA |
| **Used in RIP-PCR** |  |
| U6 Forward | CTCGCTTCGGCAGCACATATAC |
| U6 Reverse | AACGCTTCACGAATTTGCGTGTC |
| DNMT3B-L Forward | ATGGCTCTGACACCCCAGT |
| DNMT3B-L Reverse | GACTCGTCCACATGGTTGC |
| **Used in RT-PCR** |  |
| DNMT3B Forward | CAAGAGGGAGGTGTCCAGTCT |
| DNMT3B Reverse | TGTCCTCTGTGTCGTCTGTGA |
| PNPLA8 Forward | AGTGTTTGTGTTGGAAGCTCAG |
| PNPLA8 Reverse | CAGTTGCTTGCTTCTCTGCTT |
| ZDHHC7 Forward | GCAGACTTCGTGGTGACTTTC |
| ZDHHC7 Reverse | ACTTGGGGCACTTGTAGATGA |
| RREB1 Forward | TACAGAGGCACATGCTCACAC |
| RREB1 Reverse | GTCCCGTGAGGTGAGGTCTA |
| MAGI3 Forward | TCAATGGGGAACCTACACAAG |
| MAGI3 Reverse | GGGATTGATCTCTGGGTCTTC |
| SMAP Forward | CACAGACAGATCAAGCAGTGG |
| SMAP Reverse | TTTTTAGGCTCCAGTTGCTGA |
| NEK11 Forward | GTGGTTGCCCCTAGTTTGAG |
| NEK11 Reverse | CTCGTTTGGCTTTCTTGTCTG |
| PICALM Forward | AGCCTCTCCTGTATCCACCTC |
| PICALM Reverse | CTGTGCAACTGGAGAAGGAGT |
| MEIS2 Forward | GCTTGGAGGGAGTGTGCTCT |
| MEIS2 Reverse | TGAGGGTCTCCGTACATGGAA |
| PKM1 Forward | CGAGCCTCAAGTCACTCCAC |
| PKM1 Reverse | GTGAGCAGACCTGCCAGACT |
| PKM2 Forward | ATTCCGGGTCACAGCAATGATGG |
| PKM2 Reverse | ATTATTTGAGGAACTCCGCCGCCT |

**Table S8.** The probes used in the present study

| **Name** | **Sequence 5’-3’** | **Label** |
| --- | --- | --- |
| DNMT3B-L | AGAGTCGCGAGCTTGATCTT | 5’- and 3’-CY3 |
| DNMT3B-S | GAAGAGCTTTGGCATGACTG | 5’- and 3’-FAM |

**Table S9.** The oligos used in RNA pull down.

| Name | Sequence 5’-3’ | Label |
| --- | --- | --- |
| DNMT3B 4-1 | GUGGGUUACAGUCUUCCCUUCCCAGGAUAU | 5' Biotin |
| DNMT3B 4-2 | UAAACAUCAACUCUUCUAUGCAUAAUUUGG | 5' Biotin |
| DNMT3B 5-1 | GAACUGUCUUCUCCCUCAUGUCUUCUUCACU | 5' Biotin |
| DNMT3B 5-2 | CUGGAAAAGUUUCUUCAGCGGUCUCUGUUC | 5' Biotin |
| DNMT3B 5-1 Mut | GAACUGACAACUCCCUCAUGACAACUUCAC | 5' Biotin |

**Figure legends**


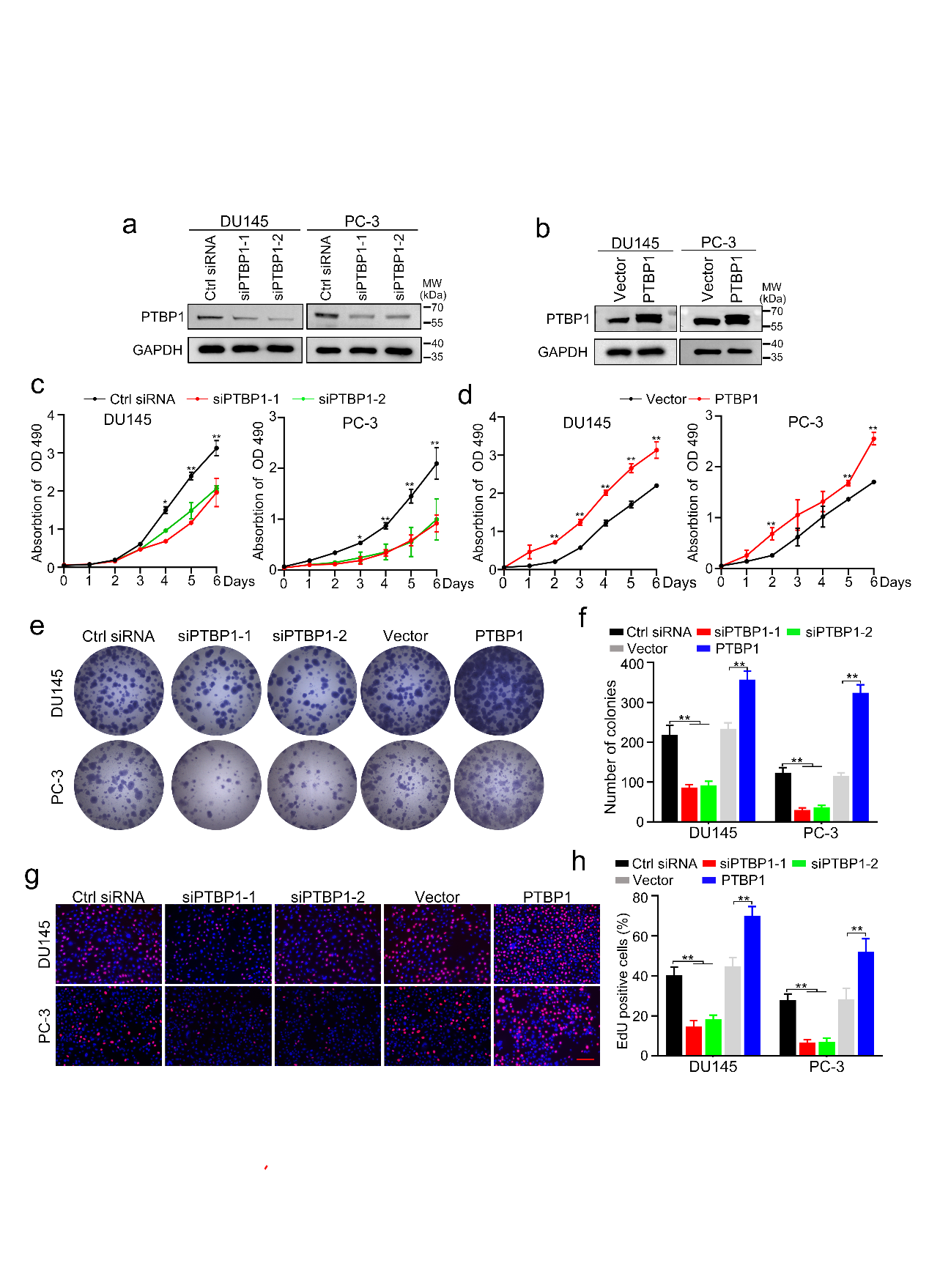
**Figure S1. PTBP1 enhances the proliferation of prostate cancer cells *in vitro*. a-b** Efficiency of PTBP1 knockdown (a) or overexpression (b) in PCa cells was confirmed by western blot. **c-d** Cell viability of PTBP1 knockdown (c) or overexpression (d) PCa cells was detected by CCK-8 assay. **e-f** Images (e) and statistical analysis (f) of colony formation assay of PTBP1 knockdown or overexpression PCa cells. **g-h** Images (g) and statistical analysis (h) of EdU assay of PTBP1 knockdown or overexpression PCa cells. Scale bar, 50μm. Statistical significance was evaluated by using a two-tailed t-test or one-way ANOVA followed by Tukey's post-hoc test where applicable. Data are presented as the mean ± S. D. of three independent experiments. **p* < 0.05, ***p* < 0.01.


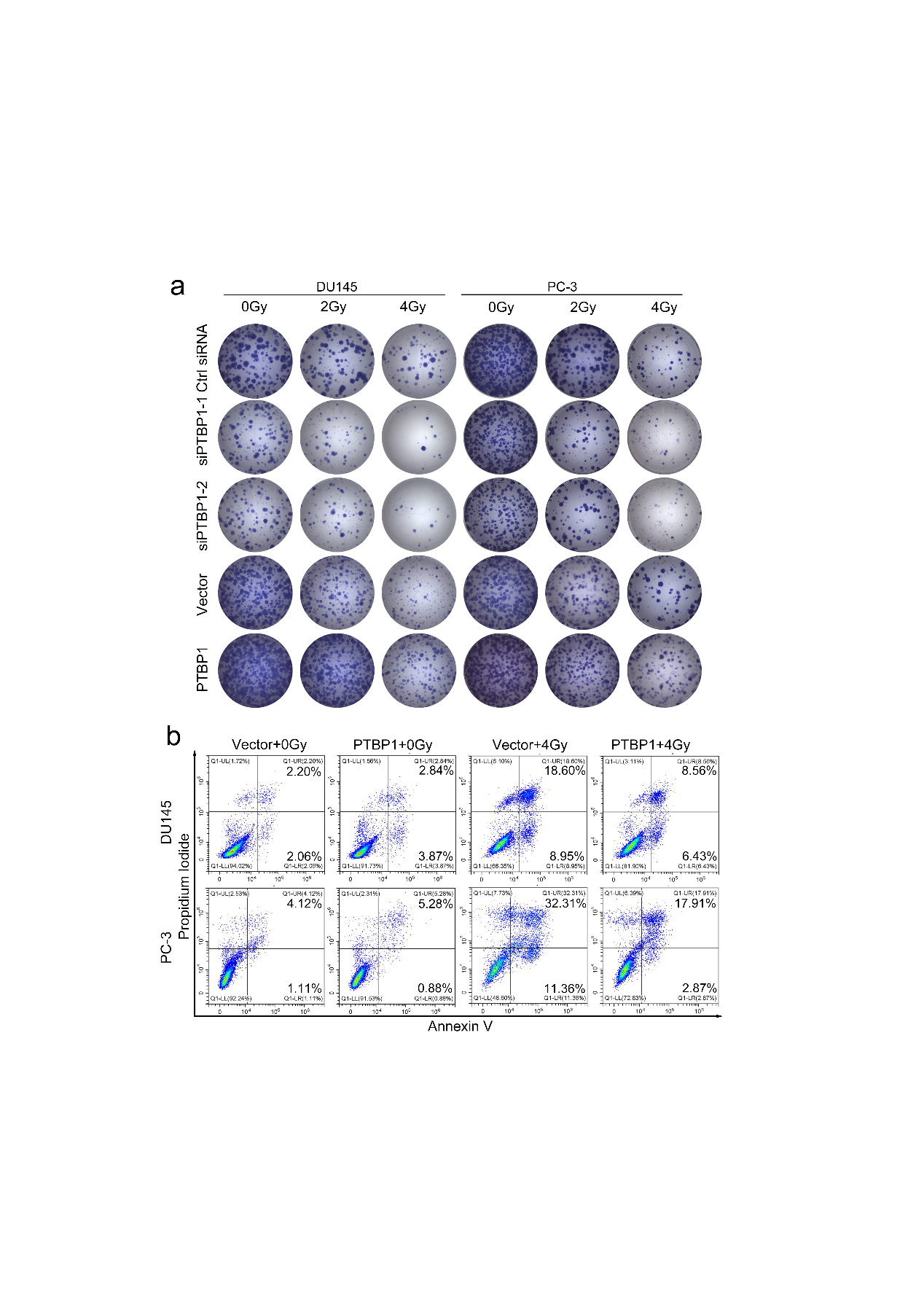
**Figure S2. PTBP1 promotes the radioresistance of prostate cancer cells *in vitro*. a** Images of colony formation assay of PTBP1 knockdown or overexpression prostate cancer (PCa) cells treated with irradiation (0, 2 and 4Gy). **b** Images of cell apoptosis of PCa cells transfected with PTBP1 or control plasmids and treated with 4Gy irradiation.


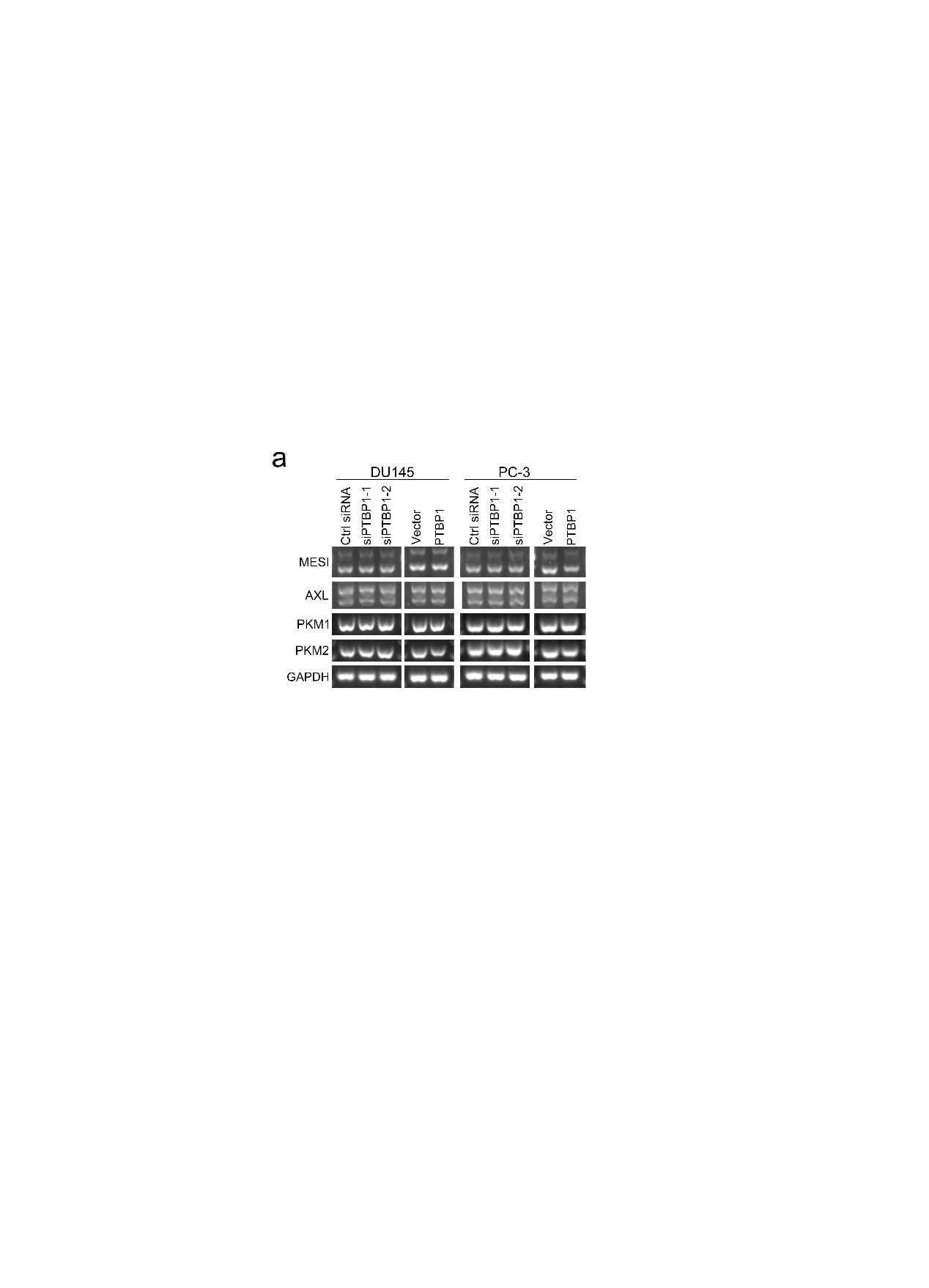


**Figure S3. PTBP1 does not regulate alternative splicing of MESI, PKM and AXL in prostate cancer cells. a** Alternative splicing of MESI, PKM and AXL in PTBP1 knockdown or overexpression prostate cancer cells were validated by agarose gel electrophoresis of PCR products.


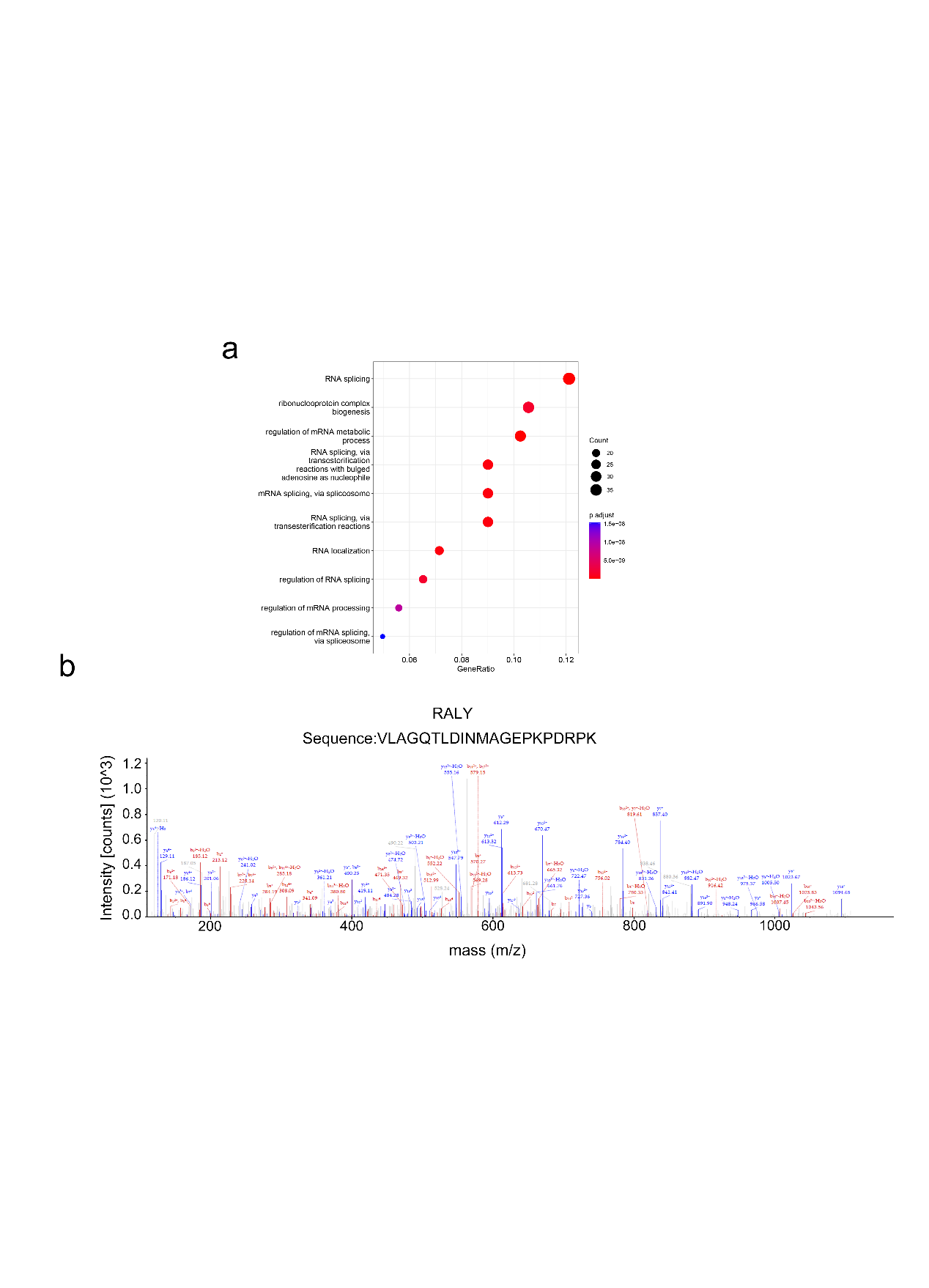


**Figure S4. PTBP1 interacts with RALY in prostate cancer cells. a** A bubble chart exhibiting the enrichment of differentially expressed genes in signaling pathways. The size and color of the bubble represent the amount of differentially expressed genes enriched in pathways and their enrichment significance, respectively. **b** Representative peptide of RALY from mass spectrometry.


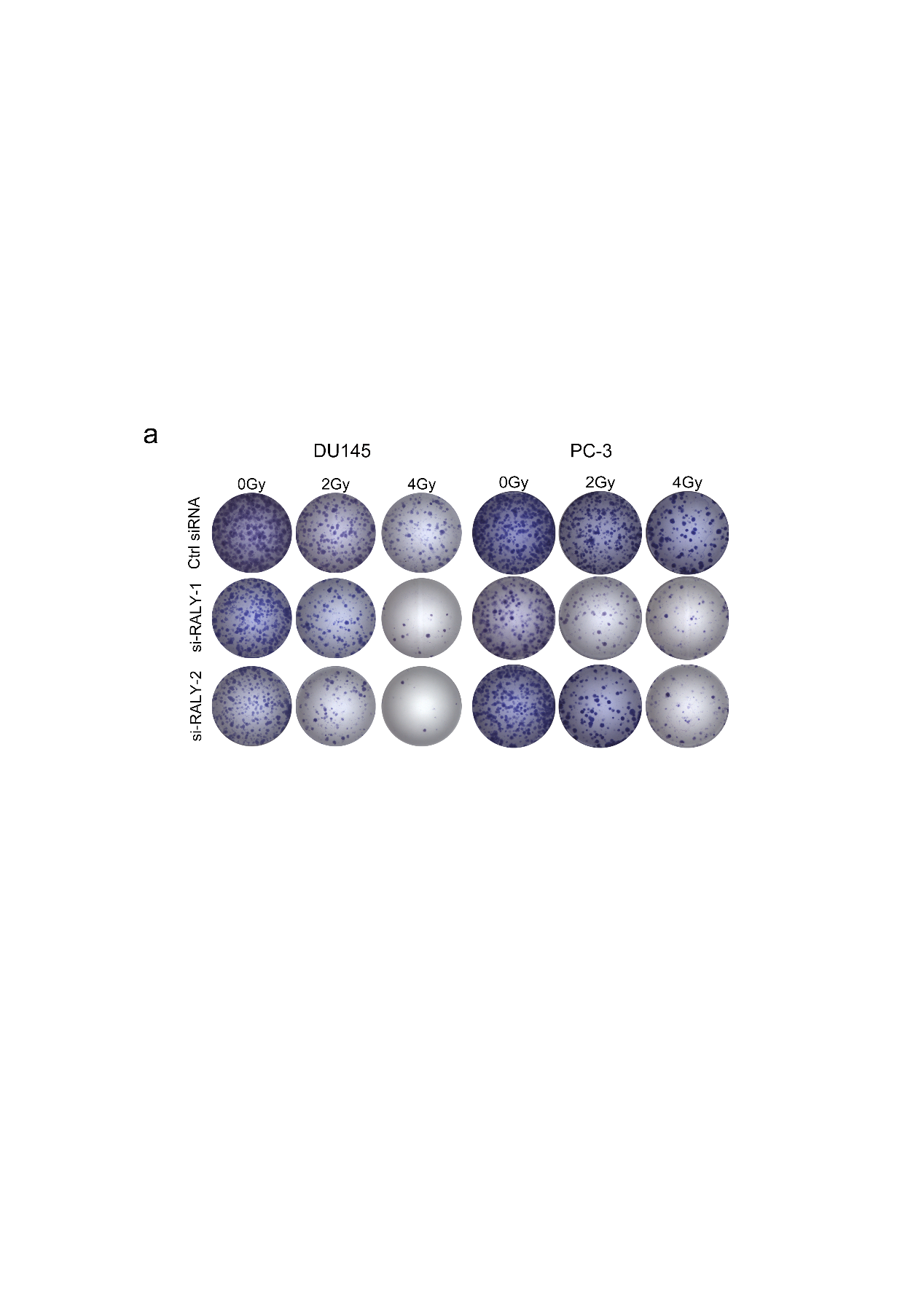


**Figure S5. RALY knockdown enhances sensitivity of** **prostate cancer cells to radiotherapy. a** Images of colony formation assay of RALY knockdown prostate cancer cells treated with irradiation (0, 2 and 4Gy).


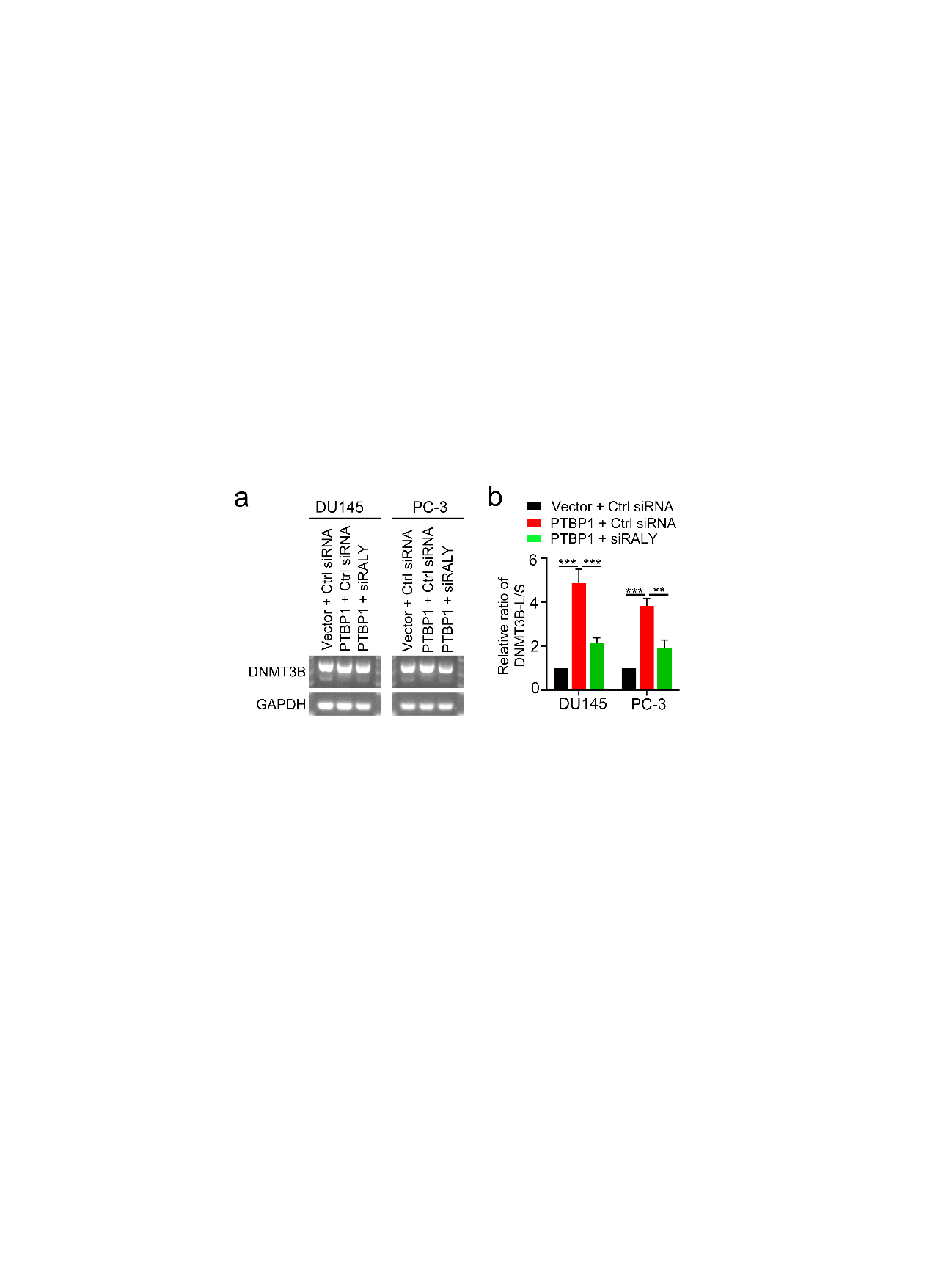


**Figure S6. RALY was involved in PTBP1-mediated alternative splicing of DNMT3B. a-b** Representative gel electrophoresis image (a) and statistical analysis (b) of the DNMT3B-L/DNMT3B-S ratio in PCa cells treated as indicated. Data are presented as the mean ± S. D. of three independent experiments. ***p*<0.01, ****p*<0.001 by two-tailed Student’s *t*-test.


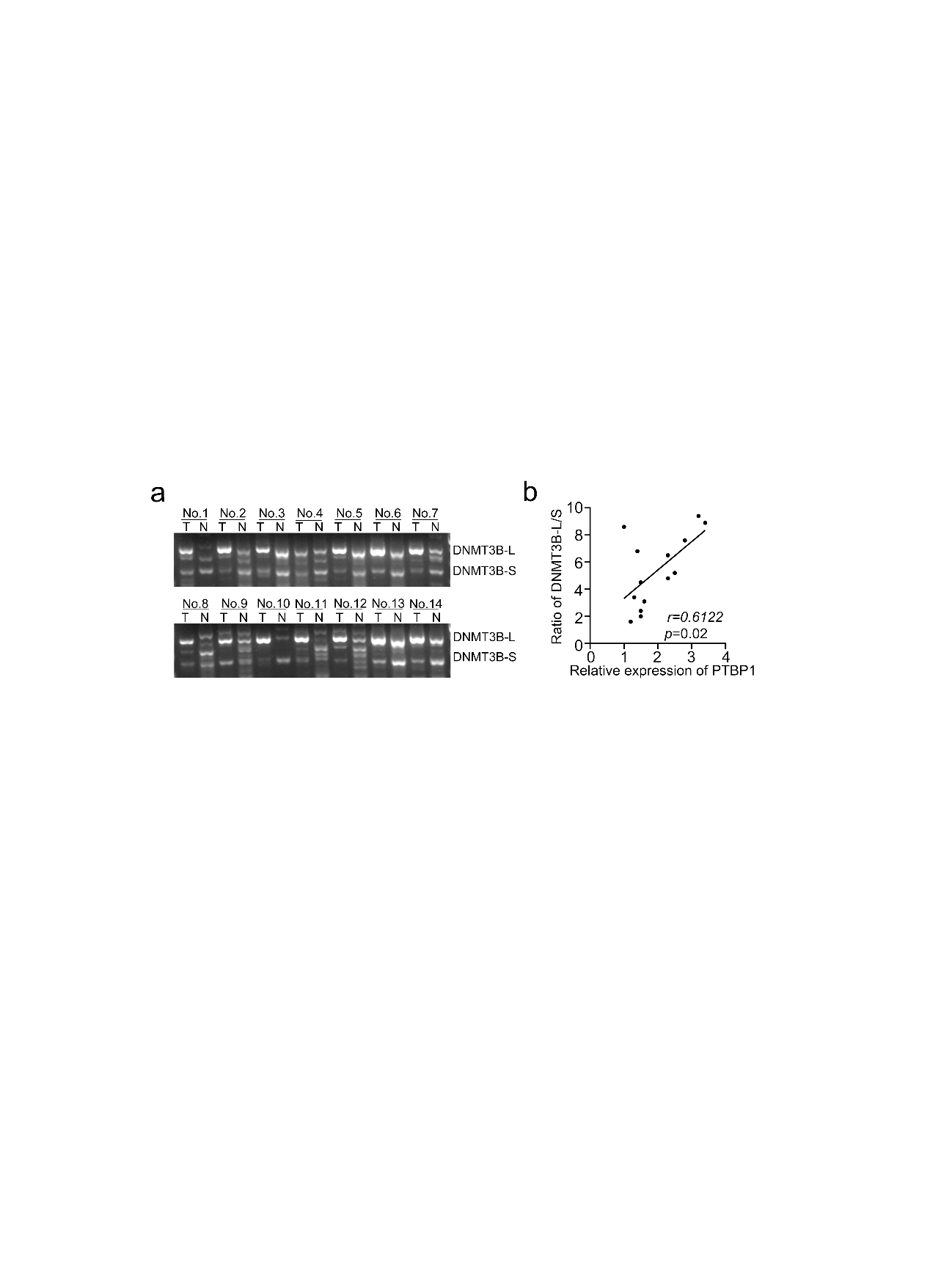


**Figure S7. DNMT3B-L was associated with PTBP1 expression in prostate cancer tissues. a** An expression of DNMT3B-L and DNMT3B-S in PCa tissues was validated by agarose gel electrophoresis of PCR products. **b** The correlation between mRNA expression of PTBP1 and DNMT3B-L/DNMT3B-S ratio in PCa tissues.


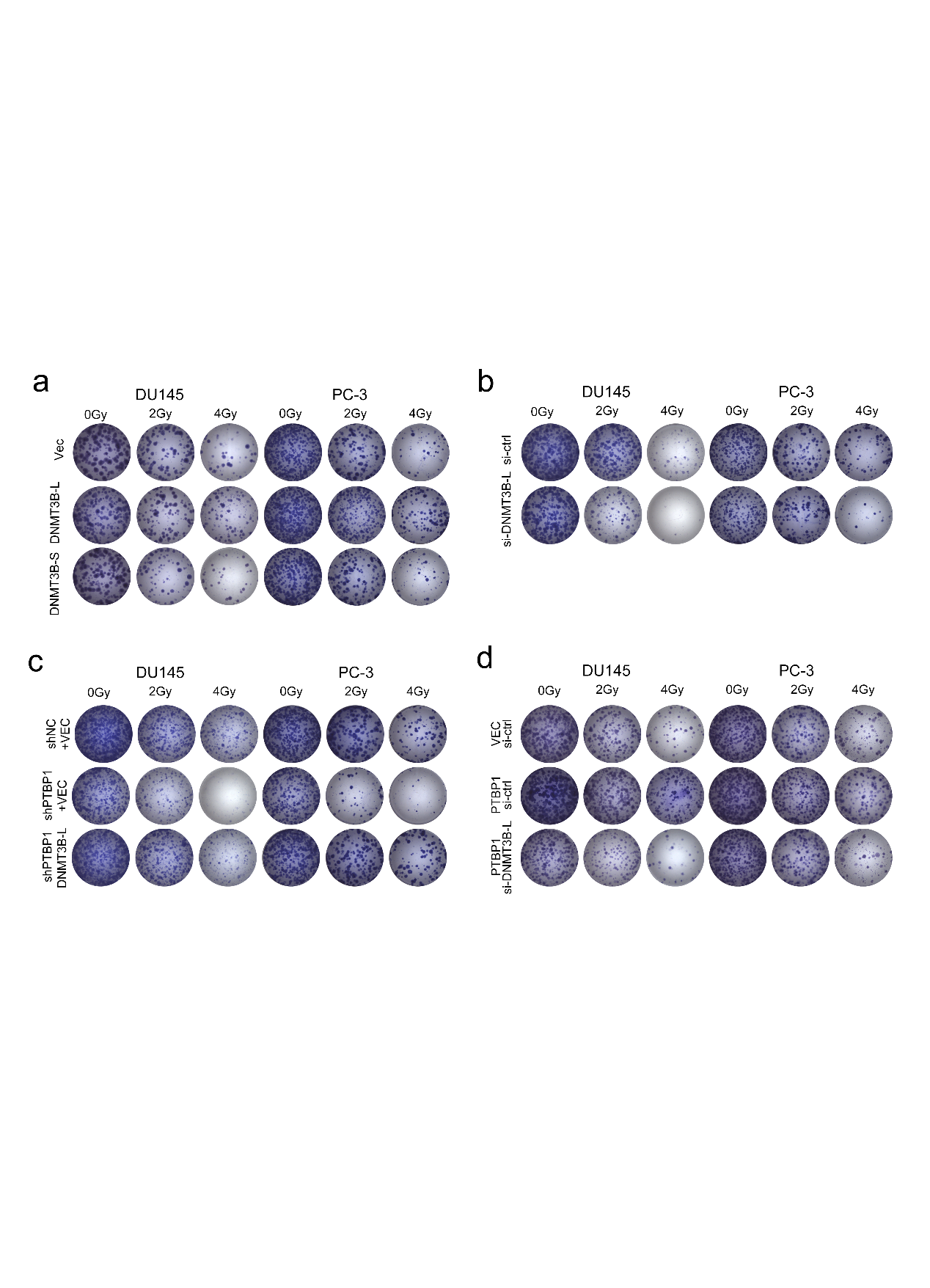


**Figure S8. PTBP1 increases radioresistance of prostate cancer cells via a DNMT3B-L dependent manner. a** Images of colony formation assay of DNMT3B-L or DNMT3B-S overexpression PCa cells treated with irradiation (0, 2 and 4Gy). **b** Images of colony formation assay of DNMT3B-L knockdown PCa cells treated with irradiation (0, 2 and 4Gy). **c-d** Images of colony formation assay of PCa cells treated as indicated following irradiation (0, 2 and 4Gy).


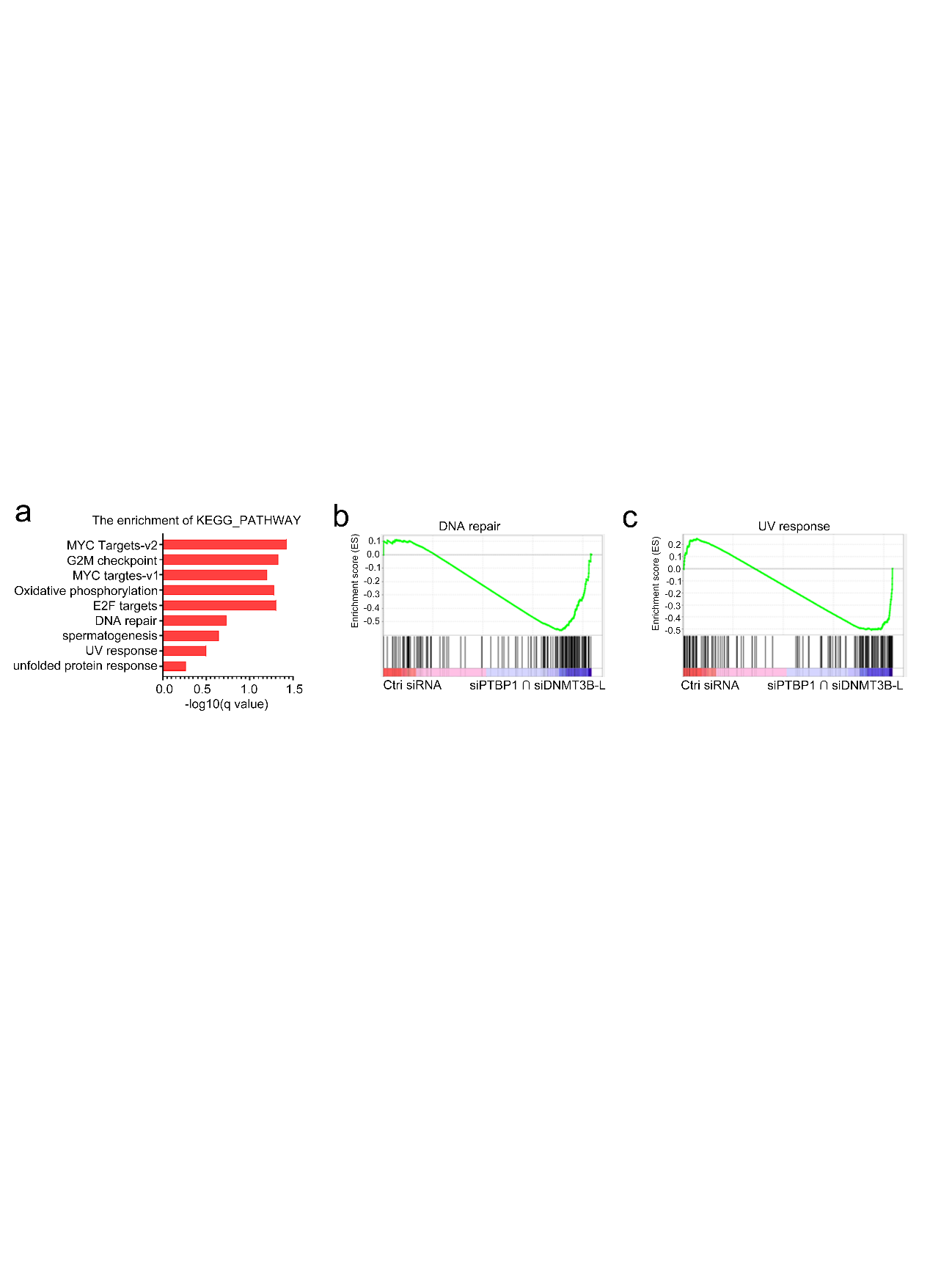
**Figure S9. Both PTBP1 and DNMT3B-L regulate DNA repair signal pathways in prostate cancer. a** Gene ontology (GO) analysis identifying the enrichment of the biological process. **b-c** Gene set enrichment analysis (GSEA) plots of DNA repair signal pathway (b) and UV response (c) signal pathway in RNA-Seq data upon silencing of PTBP1 or DNMT3B-L in DU145 cells. NES normalized enrichment score. p-values in the panel.


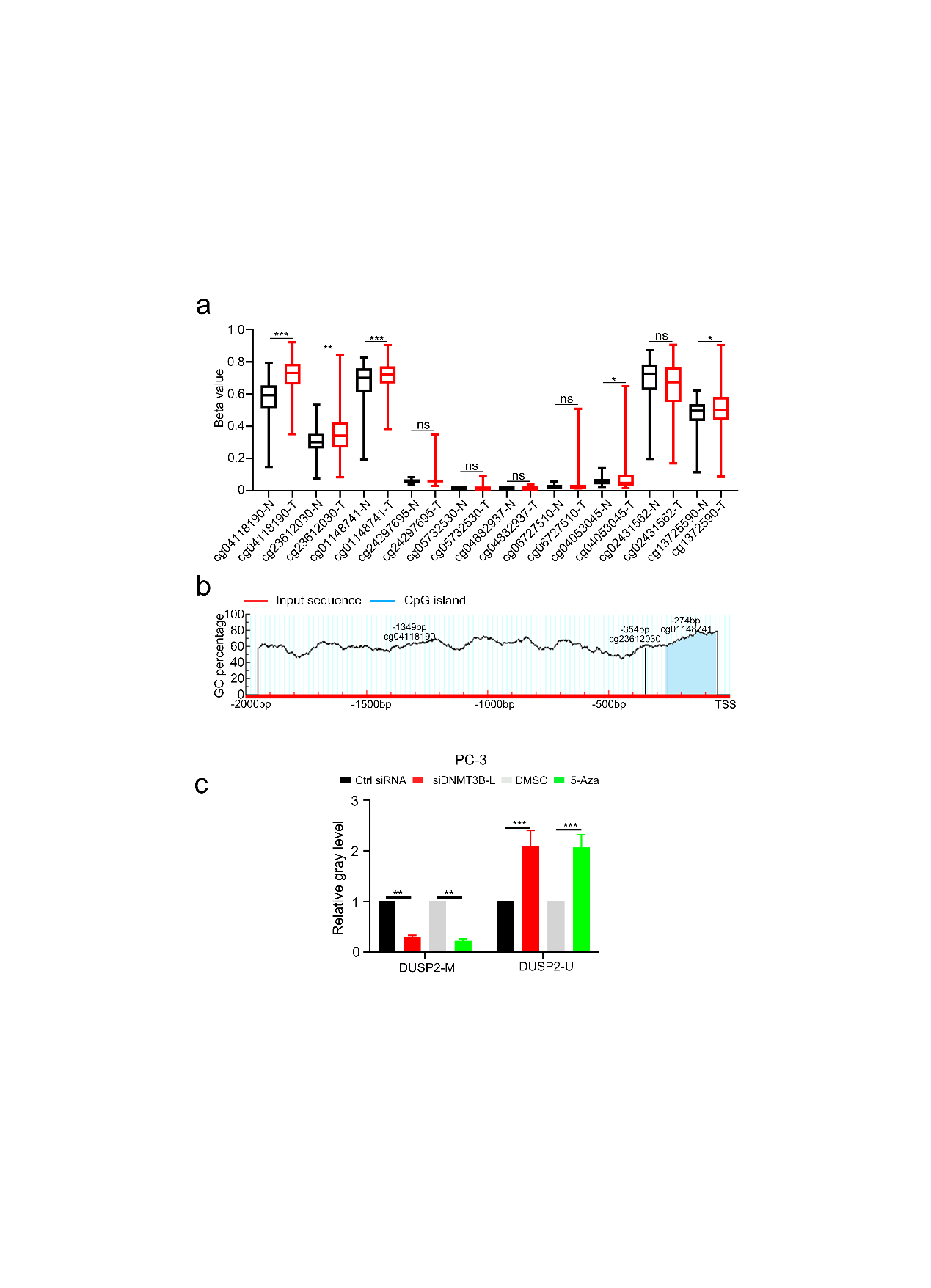


**Figure S10. The promoter of DUSP2 has high methylation in prostate cancer. a** The difference methylation in indicated methylation modification sites from DUSP2 between PCa tissues and normal tissues based on the TCGA database. **b** Schematic of the CpG islands in the DUSP2 promoter. **c** Statistical analysis of methylation of DUSP2 promoter in PC-3 cells treated as indicated. **p*<0.05, ***p*<0.01, ****p*<0.001 by two-tailed Student’s *t* test. ns. no significance.


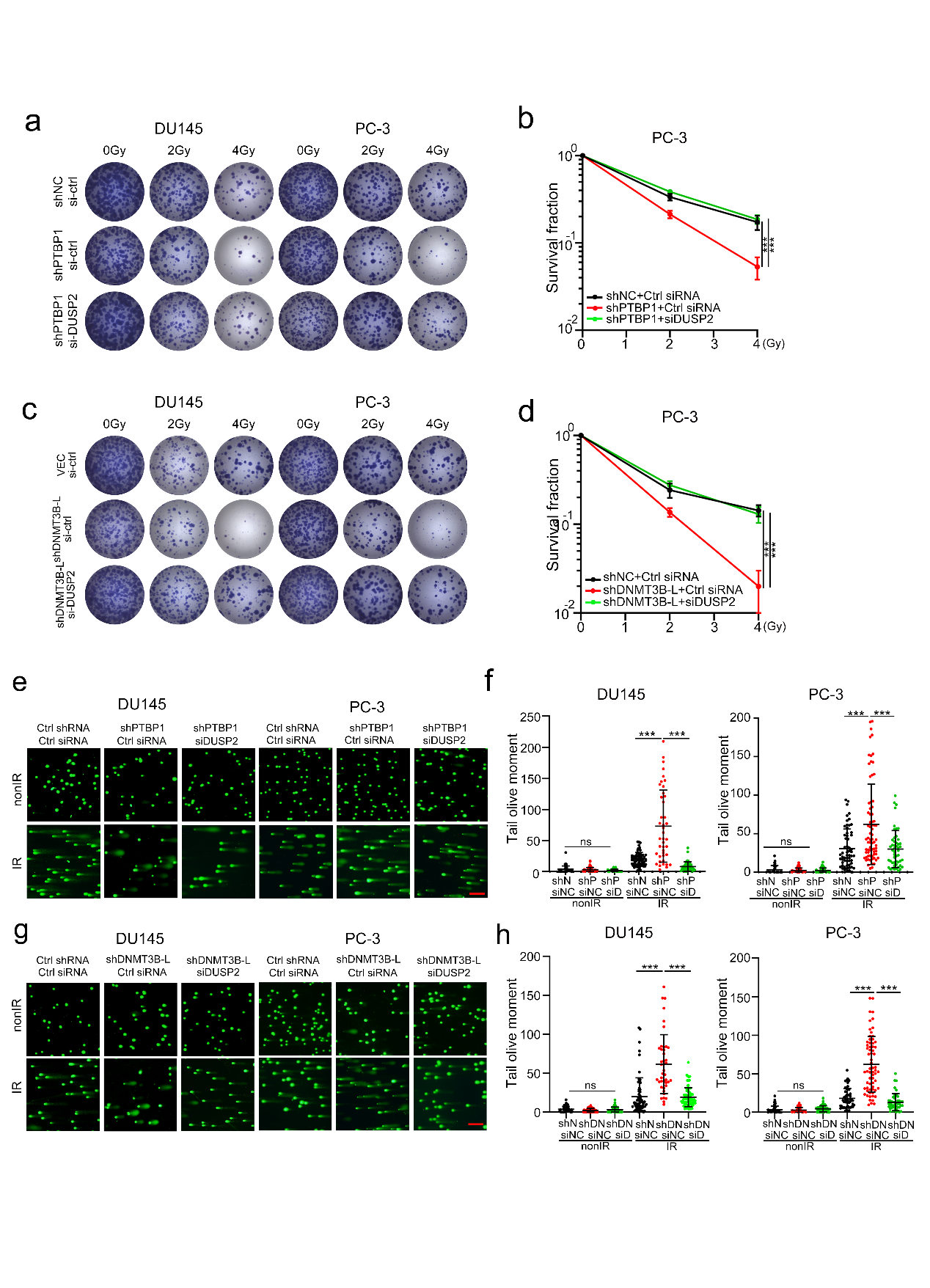


**Figure S11. DUSP2 is involved in PTBP1/DNMT3B-L-mediated radioresistance in prostate cancer. a-d** Images (a, c) and statistical analyses (b, d) of colony formation assay of PCa cells treated as indicated following irradiation (0, 2 and 4Gy). **e-h** Representative images (left) and statistical analyses (right) of comet assays of indicated DU145 (e, g) and PC-3 (f, h) cells at 24h after 4Gy irradiation. Scale bar, 20 μm. Data are presented as the mean ± S. D. of three independent experiments. **p*<0.05, ***p*<0.01, ****p*<0.001 by two-tailed Student’s *t* test. ns. no significance.
